# Effects of Antioxidant Supplementation on Metabolic Disorders in Obese Patients from Randomized Clinical Controls: A Meta-Analysis and Systematic Review

**DOI:** 10.1155/2022/7255413

**Published:** 2022-09-01

**Authors:** Jinyuan Wang, Biyun Liao, Changsheng Wang, Ou Zhong, Xiaocan Lei, Yuli Yang

**Affiliations:** ^1^Hengyang Maternal and Childe Health Hospital, Hengyang, Hunan 421001, China; ^2^Clinical Antatomy & Reproductive Medicine Application Institute, Hengyang Medical School, University of South China, Hengyan, Hunan 421001, China; ^3^Reproductive Medicine Center, The Affiliated Hospital of Youjiang Medical University for Nationalities, Baise 533000, China

## Abstract

**Purpose:**

This systematic review and meta-analysis aim at elucidating the heterogeneity in beneficial effects of antioxidant supplementation in obese adults by exploring the differential effects of antioxidant supplementation on basic indicators of obesity, lipid metabolism, systemic antioxidant capacity, inflammatory biomarkers, and liver function.

**Methods:**

The inclusion criteria specified randomized controlled trials with antioxidant intervention for adults (mean body mass index (BMI) > 30), from inception to Aug. 8, 2021, in the PubMed, Embase, The Cochrane Library, Web of Science, and Scopus databases. Meta-analysis and publication bias were performed using RevMan 5.4 software. Stata16 software was used to detect publication bias with Egger's and Begg's methods being mainly used. The data of basic indicators of obesity, lipid metabolism index, oxidative stress index, inflammatory biomarkers, and liver function index were collected to analyze the beneficial effects of antioxidant supplementation in obese patients.

**Results:**

A total of 30 studies were included in this study with a sample of 845 obese patients from the antioxidant supplementation group and 766 obese patients from the placebo control group. The meta-analysis showed that obese patients with antioxidant supplementation had lower BMI (mean difference (MD): − 0.44 [95%confidence interval (CI): − 0.84, −0.04], *p* = 0.03), waist circumference (MD : −0.78 [95%CI:−1.45, −0.11], *p* = 0.02), fasting blood glucose (FBG) level (standardized mean difference (SMD): − 4.92 [95%CI:−6.87, −2.98], *p* < 0.001) and homeostasis model assessment of insulin resistance (MD : −0.45 [95%CI:−0.61, −0.3], *p* < 0.001) when compared to the placebo group. Obese patients on antioxidant supplementation had lower levels of total cholesterol (SMD : −0.43 [95%CI:−0.84, −0.02], *p* = 0.04), triglycerides (SMD : −0.17 [95%CI:−0.31, −0.04], *p* = 0.01), low-density lipoprotein (SMD : −0.15 [95%CI:−0.29, −0.01], *p* = 0.03), malondialdehyde (SMD : −1.67 [95%CI:−2.69, −0.65], *p* = 0.001), and tumor necrosis factor-alpha (SMD : −0.29 [95%CI:−0.56, −0.02], *p* = 0.03), respectively, when compared to the placebo group. In addition, obese patients with antioxidant supplementation had higher levels of high-density lipoprotein (SMD : 0.25 [95%CI : 0.03, 0.46], *p* = 0.03) and superoxide dismutase (SMD : 1.09 [95%CI : 0.52, 1.65], *p* < 0.001) when compared to the placebo group. Antioxidant supplementation had no effects on other analyzed parameters including waist–hip ratio, leptin, fat mass, interleukin-6, C-reactive protein, alanine transaminase, and aspartate transaminase in obese patients.

**Conclusion:**

The meta-analysis results indicated that antioxidant supplementation exerted potential beneficial effects in obese patients by regulating FBG, oxidative stress, and inflammation, whilst more high-quality studies are required to confirm these effects. The present study may provide important insights for the treatment of clinical obesity and obesity-associated complications.

## 1. Introduction

With the advance of people's living standard, the global obesity epidemic has become a focus and affects health of >2 billion people [1]. Nowadays, the prevalence of obesity has doubled in about 70 countries and continues to rise [2, 3]. The occurrence of obesity impacting the body's metabolic processes, following induce serious diseases, has seriously endangering people's health [3]. Recent studies have reported a high prevalence of overweight and obesity in patients experiencing a severe COVID-19 course, with serious complications requiring hospitalization and admission to intensive care units [4]. Studies have shown that the disorder of glucose metabolism, insulin resistance and elevated blood glucose in obese patients increase the susceptibility to diabetes [5]. Dyslipidemia, increased incidence of oxidative stress and excessive production of adipocyte derivatives have all been proposed to contribute to the cardiovascular diseases [6] and non-alcoholic fatty liver disease [7] in obese patients. There are several mechanisms by which obesity produces oxidative stress. The first of these is the mitochondrial and peroxisomal oxidation of fatty acids, which can produce reactive oxygen species (ROS) in oxidation reactions, while another mechanism is over-consumption of oxygen, which generates free radicals in the mitochondrial respiratory chain that is found coupled with oxidative phosphorylation in mitochondria [8]. Lipid-rich diets are also capable of generating ROS because they can alter oxygen metabolism. Finally, high ROS production leads to various abnormalities in obese patients [8]. Adipose tissue is not only a triglyceride storage organ, but studies have shown the role of white adipose tissue as a producer of certain bioactive substances called adipokines such as interleukin-6 (IL-6), which exerts many effects, ranging from defense to inflammation and tissue damage [9]. Inflammation is a manifestation of increased oxidative, which increases in subjects with obesity, which is related with insulin resistance and endothelial dysfunction. These changes may interact among themselves and amplify, producing, in this manner, the set of metabolic and vascular alterations [10]. Therefore, in order to modify the serious impact of obesity, it is imperative to reverse the effect of obesity on oxidative stress and inflammation.

Antioxidants, such as vitamins A, C, E, selenium, zinc, copper and manganese, is a class of substances that prevent the harmful effects of ROS in daily nutrition and human health, helping capture and neutralize free radicals [11]. Antioxidants are rich in a wide range of sources, for instance green tea, strawberries, eggplant, garlic, ginger and so on, being reported possessing multiple pharmacological functions including improving the lipid metabolic abnormalities [12], insulin sensitivity, total antioxidant status [13] and anti-inflammatory [14] associated with obesity. Studies found that polyphenols found in pigmented rice may play a key role in targeting specific therapeutic pathways in obesity-related oxidative stress and inflammation [15]. Quercetin, curcumin, and resveratrol possess the antioxidant and anti-inflammatory activities, and can exert beneficial effects in obesity [16]. However, the beneficial effects of antioxidants in obese patients are still a matter of debate. A recent meta-analysis concludes that there is low-quality evidence of a beneficial effect of antioxidants to increase fertility [17]. In addition, clinical trials have reported contradictory results regarding the effect of vitamin E supplementation on weight status. Some studies reported an increase [18], some reported a decrease [19], and some reported no significant changes in body mass index (BMI) and weight following vitamin E supplementation [20]. Therefore, the effects of antioxidants on improving metabolic disorders in obese patients remain to be clarified.

In this study, we sorted out the data of basic indicators of obesity, lipid metabolism index, oxidative stress index, inflammatory biomarkers and liver function index, and aimed to clarify whether antioxidants supplementation could delay the progression of obesity and metabolic disorders in clinical application in the management of obese patients. The present study may provide important insights for the treatment of clinical obesity and obesity-associated complications.

## 2. Materials and Methods

### 2.1. Search Strategy

The electronic databases including PubMed, Embase, The Web of Science, The Cochrane library and Scopus databases were searched to collect clinical studies related to the efficacy of antioxidants supplementation in the treatment of obesity from inception to Aug 8, 2021. Articles published in peer-reviewed journals from 2006 to 2020, using a search strategy based on previous systematic reviews. Search is conducted by combining subjects and free words. The search term antioxidant∗ was added to the concept obesity. Detailed research strategy is shown in Appendix A.

### 2.2. Eligibility Criteria

Inclusion criteria: (1) Included articles were published in English, peer-reviewed, randomized and cluster randomized controlled trials (RCTs); (2) All patients had obesity (BMI ≥30 kg/m2) who did not habitually use antioxidant supplements; (3) Intervention: The treatment group received antioxidant and the control group received placebo. (3) The primary outcomes were anthropometric measurements, and secondary outcome were metabolic index. (4) Antioxidants used in RCT are biological and chemical organic nutrients that include vitamins, minerals, and polyunsaturated fatty acids, for instance, vitamin E, vitamin C, carotenoids, carnitines, coenzyme Q10 (ubiquinol), cysteine, omega-3, omega-6, the micronutrients folate, selenium, zinc, resveratrol and melatonin derived from fruits, vegetables, medicine and compound nutrient and so on [21, 22].

Exclusion criteria: (1) review articles, expert opinions, case-series/reports, basic science and conference abstracts; (2) cell experiments, animal experiments and other literatures which have unavailable data; (3) literatures with obvious statistical errors and poor quality of information; (4) literature were too old to be traced or published in other language unidentified except English. (5) smoking; alcohol consumption; the presence of pregnancy or lactation; recent surgery, patients with coronary heart disease; known cases of diabetes mellitus; patients with proven malignancy, chronic kidney or hepatic disease, asthma, chronic cough, chronic inflammatory disease and psychological problems; obesity due to endocrine disease (hypothyroidism or uncontrolled thyroid disease); and genetic obesity syndrome. or history of use of any dietary supplements in the 3 months prior to the study.

### 2.3. Literature Screening and Data Extraction

After literature retrieval, eliminating duplicate literatures, we carried out a relevance check of 10644 articles. By following thee inclusion and exclusion criteria, at least two reviewers independently screened the title, abstract and full text of the articles using a data collection form. After excluding the literatures with obviously irrelevant contents, we further read the full text. According to the inclusion and exclusion criteria, the included literatures were identified and then the data was extracted. The eligibility of the studies was assessed by two independent reviewers and discrepancy was resolved with double checking the data by either: (a) discussion and consensus or (b) by a third independent reviewer. Using Cohen's kappa statistic, the overall agreement rate prior to correcting discrepancies is shown in Table 1.

### 2.4. Statistical Analysis

Statistical meta-analyses of pooled effect and heterogeneity index (I^2^) as well as forest and funnel plots were carried out using the RevMan 5.3 software. For assessing the reliability of the data extraction process, two reviewers independently screen and select the RCT, coding the characteristics of all studies that fulfilled the selection criteria. Continuous data were estimated with weighted mean difference (MD) and confidence intervals (CIs) were set at 95%, and *p* < 0.05 was considered statistically significant. For the sake of eliminating the influence of different units, standardized mean difference (SMD) was used for assessment (Table 2). If *I*^2^ < 50% or *p* > 0.1, which indicated that little heterogeneity among the included studies, the fixed effect model was used; on the contrary, the significant heterogeneity among the included studies result in the random effects model adopted. Egger's and Begg's tests were mainly performed by Stata16 software in order to detect publication bias, *p* > 0.05 indicates no significant publication bias (when the *p* values of Egger's and Begg's tests are contradictory, the Egger's examination results are more convincing).

## 3. Results

### 3.1. Study Selection

**Figure 1:**
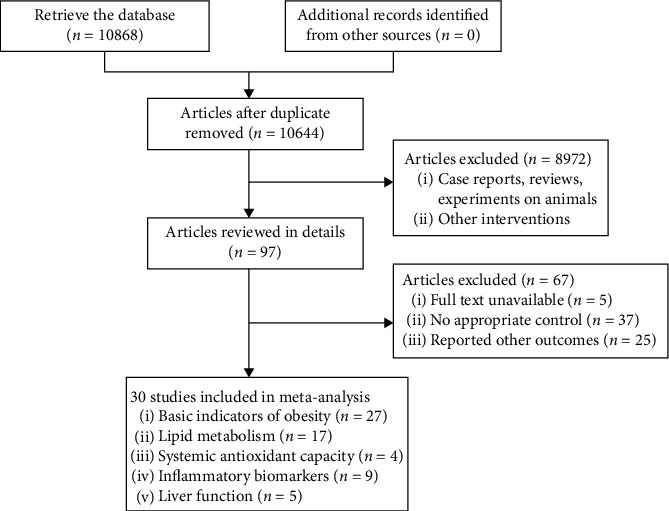
Flowchart of study selection.

We identified 10,868 literatures in the initial retrieval, including PubMed (*n* = 465), Embase (*n* = 341), The Cochrane library (*n* = 4471), Web of Science (*n* = 4675), and Scopus (*n* = 921). After carefully reviewing the titles and abstracts of these literatures, 224 duplicate articles were excluded. After further screening, 30 studies were included in this meta-analysis, the literature screening process and results are shown in Figure 1. We evaluated RCTs from 30 studies and found a total of 27 trials reporting basic indicators of obesity, 17 trials reporting lipid metabolism, 4 trials reporting systemic antioxidant capacity, 9 trials reporting inflammatory biomarkers, and 5 trials reporting liver function (Figure 1).

### 3.2. Study Characteristics

A total of 30 individual RCTs were eventually included in this study, and 93.33% of RCTs ended the interventions by 6 to 15 weeks. The demographics of the patients were summarized in Table 3 (placebo group: 845 patients; antioxidants supplementation group: 766 patients). The quality of the included articles was evaluated using the bias risk assessment tool for RCT in Cochrane Systematic Review Manual 5.1.0, and all the included studies reached a medium to high level. However, some studies did not describe the method of random assignment of included cases, and did not describe whether the assignment was hidden (Table 4). Thus, we assessed the study quality using the bias risk plots, and evaluation results of bias risk are shown in Supplemental Figure S1.

### 3.3. Effects of Antioxidant Supplementation on Basic Indicators of Obesity in Obese Patients

**Figure 2:**
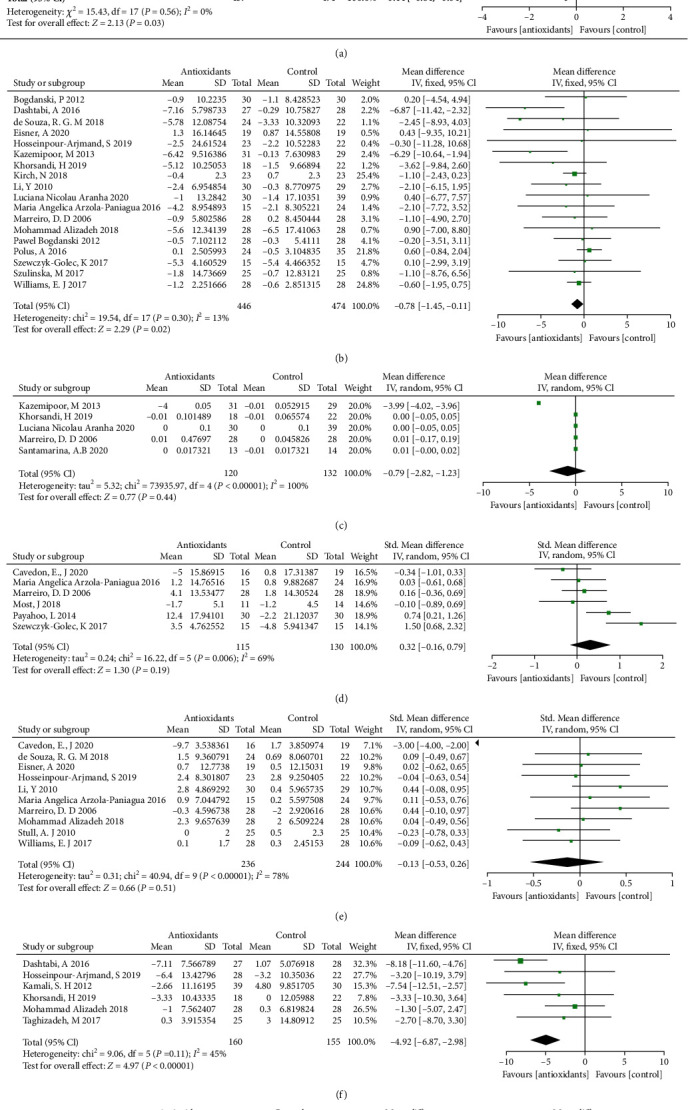
Forest plot evaluating the effects of antioxidants on basic indicators of obesity: BMI (a), WC (b), WHR (c), leptin (d), FM (e), FBG (f), and HOMA-ir (g) in obesity patients and compared with the control group.

The forest plots from stratified meta-analyses in Figure 2 depicted a total of 27 study outcomes, which reported the effects of antioxidants supplementation on basic indicators of obesity. The results showed that obese patients with antioxidants supplementation had lower BMI (*p* = 0.03; Figure 2(a)), waist circumference (WC; *p* = 0.02; Figure 2(b)), fasting blood glucose (FBG) level (*p* < 0.001; Figure 2(f)) and homeostatic model assessment for insulin resistance (HOMA-IR; *p* < 0.001; Figure 2(g)), respectively, when compared to the placebo group. While there was no statistical significance in waist–hip ratio (WHR; *p* = 0.44; Figure 2(c)), leptin level (*p* = 0.19; Figure 2(d)) and fat mass (FM; *p* = 0.51; Figure 2(e)) between placebo control and antioxidants treatment group. Begg's test and Egger's test showed no significant publication bias for BMI (*p*_(B)_ = 1.4943, *p*_(E)_ = 0.8978), WC (*p*_(B)_ = 1.7225, *p*_(E)_ = 0.7412), FBG (*p*_(B)_ = 1.5476, *p*_(E)_ = 0.9121) and HOMA-IR (*p*_(B)_ = 0.9015, *p*_(E)_ = 0.0629). The corresponding funnel plots for evaluating the publication bias are shown in Supplemental Figure S2. The subgroup analysis showed that obese patients with water-soluble antioxidants supplementation had lower WC (*p* <0.01), FBG (*p* < 0.001) and HOMA-IR (*p* < 0.001) when compared to the placebo group (Supplementary Table S1). Obese patients with fat-soluble antioxidants supplementation had higher leptin level when compared to the placebo group (*p* < 0.05; Supplementary Table S1). However, no significant difference was detected in the basic indicators of obesity between mixed antioxidants treatment group and placebo control group (*p* > 0.05; Supplementary Table S1).

### 3.4. Effects of Antioxidant Supplementation on Regulating Lipid Metabolism in Obese Patients

**Figure 3:**
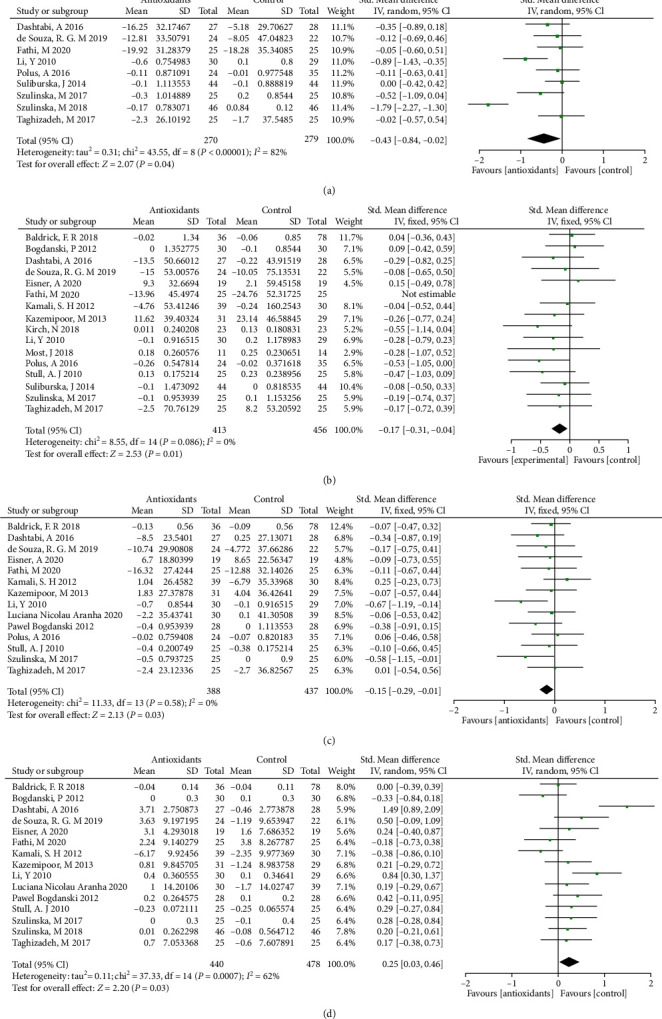
Forest plot evaluating the effects of antioxidants on lipid metabolism indexes: TC (a), TG (b), LDL (c), and HDL (d) in obesity patients and compared with the control group.

The forest plots from stratified meta-analyses in Figure 3 depicted a total of 17 study outcomes, which reported the effects of antioxidants on regulating lipid metabolism in obese patients. The results showed that obese patients on antioxidants supplementation had lower total cholesterol (TC) level (*p* = 0.04; Figure 3(a)), triglycerides (TG) level (*p* = 0.01; Figure 3(b)) and low-density lipoprotein (LDL) level (*p* = 0.03; Figure 3(c)), respectively, when compared to the placebo group. The high-density lipoprotein (HDL) level in antioxidants supplementation group was higher than placebo group (*p* = 0.03; Figure 3(d)). Begg's test and Egger's test showed no significant publication bias for TC (*p*_(B)_ = 1.2455, *p*_(E)_ = 0.7151), TG (*p*_(B)_ = 1.6996, *p*_(E)_ = 0.3787), LDL (*p*_(B)_ = 1.8747, *p*_(E)_ = 0.3505), and HDL (*p*_(B)_ = 0.0377, *p*_(E)_ = 0.1254). The corresponding funnel plots for evaluating the publication bias are shown in Supplemental Figure S3. In the subgroup analysis, no significant difference was detected in the TC, TG, LDL and HDC between water-soluble antioxidants treatment group and placebo control group (*p* > 0.05; Supplementary Table S2), and similar results were also detected in the fat-soluble antioxidants subgroup (*p* > 0.05; Supplementary Table S2). Furthermore, obese patients with mixed antioxidants supplementation had higher HDL when compared to the placebo group (*p* < 0.001; Supplementary Table S2).

### 3.5. Effects of Antioxidant Supplementation on Systemic Antioxidant Capacity in Obese Patients

**Figure 4:**
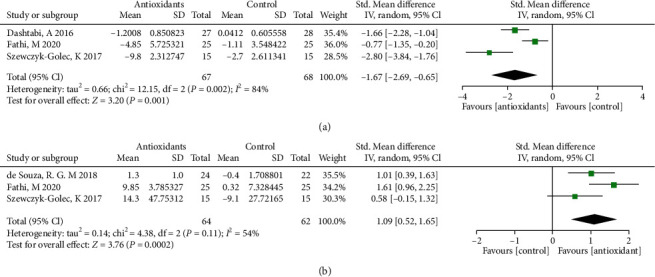
Forest plot evaluating the effects of antioxidants on systemic antioxidant capacity indexes: MDA (a) and SOD (b) in obesity patients and compared with the control group.

The forest plots from stratified meta-analyses in Figure 4 depicted a total of 4 study outcomes, which reported the effects of antioxidants on regulating systemic antioxidant capacity in obese patients. The results showed that obese patients with antioxidants supplementation had lower malondialdehyde (MDA) level (*p* = 0.001; Figure 4(a)) when compared to placebo group. The superoxide dismutase (SOD) level in antioxidants supplementation group was higher than in the placebo group (*p* < 0.001; Figure 4(b)). Begg's test and Egger's test showed no significant publication bias for MDA (*p*_(B)_ = 1.9633, *p*_(E)_ = 0.2680), and SOD (*p*_(B)_ = 1.7037, *p*_(E)_ = 0.5561). The corresponding funnel plots for evaluating the publication bias are shown in Supplemental Figure S4. The subgroup analysis showed that obese patients with water-soluble antioxidants supplementation had lower MDA level (*p* < 0.01) and higher SOD level (*p* < 0.001) when compared to the placebo group (Supplementary Table S3). Obese patients with fat-soluble antioxidants supplementation had lower MDA level when compared to the placebo group (*p* < 0.001; Supplementary Table S3). In addition, obese patients with mixed antioxidants supplementation had higher SOD level when compared to the placebo group (*p* < 0.05; Supplementary Table S3).

### 3.6. Effects of Antioxidant Supplementation on Levels of Inflammatory Markers in Obese Patients

**Figure 5:**
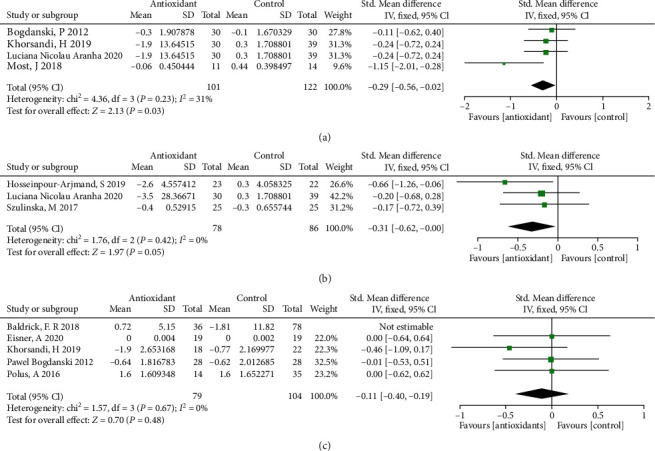
Forest plot evaluating the effects of antioxidants on inflammatory biomarkers: TNF-*α* (a), IL-6 (b), and CRP (c) in obesity patients and compared with the control group.

The forest plots from stratified meta-analyses in Figure 4 depicted a total of 4 study outcomes, which reported the effects of antioxidants on systemic antioxidant capacity in obese patients. The results showed that obese patients with antioxidants supplementation had lower tumor necrosis factor-alpha (TNF-*α*) level (*p* = 0.03; Figure 5(a)) when compared to the placebo group. However, antioxidants supplementation in the obese patients did not affect the levels of IL-6 (*p* = 0.05; Figure 5(b)) and C-reactive protein (CRP; *p* = 0.86; Figure 5(c)) when compared to the placebo group. Begg's test and Egger's test showed no significant publication bias for TNF-*α* (*p*_(B)_ = 1.5558), *p*_(E)_ = 0.1752). The corresponding funnel plots for evaluating the publication bias are shown in Supplemental Figure S5. The subgroup analysis showed that obese patients with water-soluble antioxidants supplementation had lower TNF-*α* level (*p* < 0.05) when compared to the placebo group (Supplementary Table S4). Obese patients with fat-soluble antioxidants supplementation had lower IL-6 level when compared to the placebo group (*p* < 0.05; Supplementary Table S4). However, no significant difference was detected in inflammatory markers between mixed antioxidants treatment group and placebo control group (*p* > 0.05; Supplementary Table S4).

### 3.7. Effects of Antioxidant Supplementation on Liver Function in Obese Patients

**Figure 6:**
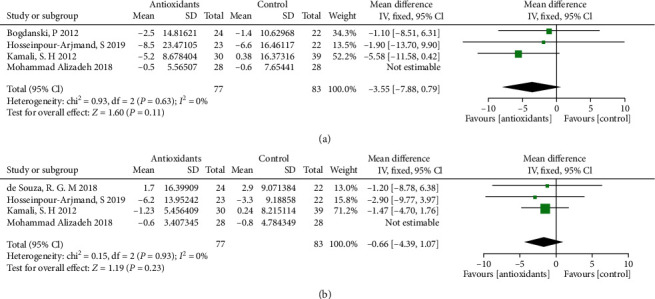
Forest plot evaluating the effects of antioxidants on liver function indexes: ALT (a) and AST (b) obesity patients and compared with the control group.

The forest plots from stratified meta-analyses in Figure 5 depicted a total of 5 study outcomes, which reported the effects of antioxidants on liver function in obese patients. The results showed that antioxidants supplementation in obese patients did not affect the levels of alanine transaminase (ALT; *p* = 0.34; Figure 6(a)) and aspartate transaminase (AST; *p* = 0.55; Figure 6(b)) when compared to the placebo group. Begg's test and Egger's test showed no significant publication bias for AST (*p*_(B)_ = 1.6918, *p*_(E)_ = 0.9968) and ALT (*p*_(B)_ = 1.2659, *p*_(E)_ = 0.4782). The corresponding funnel plots for evaluating the publication bias are shown in Supplemental Figure S6. The subgroup analysis further revealed that obese patients with water-soluble, fat-soluble, or mixed antioxidants supplementation did not exhibit any significant changes in the levels of ALT (*p* > 0.05) and AST (*p* > 0.05) when compared to the placebo group (Supplementary Table S5).

## 4. Discussion

Obesity, which considered a global health problem, has met the medical definition of disease. Complications from obesity affect almost every tissue in the body, which is a major cause of cardiovascular events, diabetes [23] as well as infertility [24] and so on. Obesity triggers a host of metabolic disorders, while there are no effective treatments for it. Diet-derived antioxidant, a class of substances that prevent the harmful effects of ROS in daily nutrition and human health, helping capture and neutralize free radicals, has plentiful positive effects on human metabolism [11]. Moreover, several different kinds of antioxidants are reported to improve the metabolic abnormalities associated with obesity [12–14] . However, the effects of antioxidants on improving metabolic disorders in obese patients are controversial. In this meta-analysis, we further explored the effects of antioxidants on obesity in terms of basic indicators of obesity, lipid metabolism, systemic antioxidant capacity, inflammatory biomarkers and liver function, and further examined whether antioxidants can effectively improve the metabolic function in obese patients, which may provide robust evidence for clinical obesity management.

Firstly, we collected data from 27 studies to assess the effects of antioxidants supplementation on improving physiological dimension in obese patients. The results of meta-analysis showed that antioxidants supplementation could significantly decrease BMI and WC level when compared to the placebo group. Previous studies also showed that anthocyanin (possessing antioxidant properties) supplementation was sufficient to reduce the BMI and body weight in obese patients [25]. However, green tea extract as antioxidants supplementation was not associated with reductions in BMI or WC in obese women [26]. However, our studies failed to prove that antioxidants have a beneficial effect on WHR and FM levels in patients. Leptin as major adipokine and the product of obese gene, synthesized by the white adipocyte tissue, mastering feeding and metabolism acting at central level in the brain [27]. Increased leptin levels were found in obese animal [28]. Leptin could improve insulin sensitivity and reduce insulin resistance [29]. Our result showed that antioxidants supplementation did not significantly affect leptin level in the obese patient, which was consistent with previous findings showing that neither green tea intake nor resveratrol intake had significant effects on serum levels of leptin [30, 31]. On the other hand, antioxidants supplementation significantly decreased HOMA-IR and FBG value in obesity patients. Studies have shown that 60-day *saccharomyces boulardii* and superoxide Dismutase supplementation could decrease HOMA-IR in obese adults [32]. Ellulu et al. demonstrate that Vitamin C (500 mg twice daily) could reduce FBG in hypertensive and/or diabetic obese patients [33]. Collectively, the effect of antioxidants on basic indicators and glucose metabolic function with obesity was positive.

Obesity is a common disease, which is the manifestation of excess body fat and is closely associated with the disorder of lipid metabolism, which driven by the effects of insulin resistance and pro-inflammatory adipokines [34]. The disorder of lipid metabolism in obese patients is manifested as high TC, TG and LDL levels and low HDL levels [35]. High-fat diet causes the imbalance between lipid absorption and metabolism, resulting in lipid metabolism disorders, which can cause a variety of metabolic diseases such as cardiovascular and cerebrovascular events and non-alcoholic fatty liver [36]. However, antioxidants do modulate lipid metabolism in the animal studies and RCTs [37, 38]. Therefore, we collected data from 17 existing studies to evaluate the role antioxidants played in the treatment of dyslipidemia with obesity. The meta-analysis results revealed that that antioxidants supplementation could reduce TC, TG and LDL levels and increased HDL levels in obese patients when compared to the placebo group, which was consistent with previous findings [39, 40], However, the Begg's test suggests the publication bias in these included studies, thus, the effects of antioxidants on regulating lipid metabolism disorder require further confirmation by more studies.

The high fat diet and carbohydrates can cause a significant increase in oxidative stress and inflammation in obese patients [41]. MDA as a oxidative stress biomarker, is the products of the peroxidation of polyunsaturated fatty acids, and is elevated in the serum of obese human and animals [42, 43]. While with increasing of adipose tissue, the activity of antioxidant enzymes such as SOD diminished significantly [8]. Those above evidence suggests that systemic antioxidant capacity was impaired in obese patients, while, antioxidants supplementation has been found to alleviate the impairment in obese patients [44]. In this study, we collected data from 4 existing studies to evaluate the importance of antioxidants in the ameliorating systemic antioxidant capacity with obesity. Results of meta-analysis revealed that antioxidants supplementation reduced MDA level and increased SOD level in obese patients when compared to the placebo group. However, existing RCTs rarely mention changes in other oxidative stress markers such as catalase, glutathione, peroxidase, total antioxidant capacity and so on, therefore, these effects of antioxidant supplementation on these parameters have not been examined in this study. The above evidence indicated the beneficial effects of antioxidants on oxidative stress in obese patients; whilst more high-quality studies are still required to confirm the results from this meta-analysis.

Inflammation is also a common pathological process in obese patients [41]. Dysfunctional adipocytes can secrete inflammatory adipokines such as such as TNF-*α* and IL-6, which can initiate adipose tissue inflammation [45]. The occurrence of adipose tissue inflammation in different tissues can negatively impacts organ function, for example reduced oocyte quality [46] and cancer [47]. Besides, CRP is also increased in obese patients as proinflammatory biomarkers. In this study, results of meta-analysis indicated that antioxidants supplementation in obese patients significantly reduced TNF-*α* level when compared to the placebo group, while the IL-6 and CRP levels were not significantly affected by antioxidants supplementation. These results suggest that antioxidants supplementation may attenuated inflammation in obese patients, which may be confirmed by more high-quality studies.

Accumulation of body fat and abnormal lipid metabolism in obese patients can seriously affect liver function [48, 49]. AST and ALT are mainly synthesized in the mitochondria of liver cells, and elevation of AST and ALT levels are closely correlated with impaired liver function. Animal studies have shown that antioxidants can improve nonalcoholic fatty liver disease in mice with high fat diet-induced obesity [50]. In this study, we collected data from 5 existing studies to evaluate the importance of antioxidants in liver function of obese patients. Our meta-analysis results showed that antioxidants supplementation in obese patients had no effect on AST and ALT levels; while the effects of antioxidants on the liver function of obese patients still require further investigation.

Antioxidants have different chemical structures and can be classified into two gross divisions, depending on their solubility in water (hydrophilic) or fat (hydrophobic). Generally, water soluble antioxidants react with ROS within cells or body fluids (blood serum, extracellular fluid, seminal plasma) while fat-soluble antioxidants are more prone to protect cell membranes from ROS-mediated lipid peroxidation [51, 52]. Lazzarino et al., showed that fat-soluble antioxidants in seminal plasma was much lower than water-soluble antioxidants, suggesting that their administration to treat male infertility characterized by excess ROS production should be performed for a prolonged period of time [51]. Our subgroup analysis showed that different types of antioxidants had differential effects on metabolic disorders, while the specific roles of water- and fat-soluble antioxidants in obese patients may still require further studies.

The strengthen of the study is that the meta-analysis is the first article to study the effects of clinical antioxidants on the metabolism of obesity, providing a therapeutic basis for clinical intervention in obesity-induced metabolic disorders. This systematic review has the following limitations: (1) studies, study populations, and main results in cumulative analyses were heterogeneous. Due to the relatively small number of studies, our analyses had limited power. (2) Only English paper was took into account in this study, which may affect the accuracy of the results. (3) The types of antioxidants included in the study were diverse. Thus, it was not clear which antioxidant was the most effective. In addition, for more precise findings and accurate conclusions, more high-quality trials are needed to assess the beneficial effects of antioxidants on obesity and its metabolism. (4) The protocol of this study has not been pre-registered, which may induce potential bias to the review.

## 5. Conclusions

The meta-analysis results indicated that antioxidants supplementation exerted potential beneficial effects in obese patients by regulating FBG, oxidative stress and inflammation, whilst more high-quality studies are required to confirm these effects. The present study may provide important insights for the treatment of clinical obesity and obesity-associated complications.

## Figures and Tables

**Table 1:** Cohen's kappa statistics between the authors of clinical outcomes.

Outcomes	The interrater reliability		The intrarater reliability	
	Pooled *k*	95% confidence interval (CI)	Pooled *k*	95% CI
BMI (kg/m^2^)	0.62	(0.52, 0.70)	0.60	(0.55, 0.69)
WC (cm)	0.62	(0.54, 0.69)	0.59	(0.52, 0.69)
WHR (mmol/L)	0.65	(0.57, 0.73)	0.61	(0.53, 0.72)
Leptin (*μ*g/L)	0.66	(0.54, 0.72)	0.62	(0.51, 0.68)
FM (kg)	0.55	(0.42, 0.63)	0.58	(0.51, 0.65)
FBG (*μ*g/dL)	0.60	(0.52, 0.67)	0.61	(0.53, 0.72)
HOMAir	0.57	(0.50, 0.66)	0.59	(0.51, 0.69)
TC (*μ*g/dL)	0.61	(0.54, 0.72)	0.59	(0.50, 0.72)
TG (*μ*g/dL)	0.59	(0.52, 0.69)	0.60	(0.52, 0.72)
LDL (*μ*g/dL)	0.63	(0.55, 0.70)	0.60	(0.54, 0.71)
HDL (*μ*g/dL)	0.65	(0.53, 0.67)	0.61	(0.51, 0.70)
MDA (mmol/L)	0.66	(0.60, 0.74)	0.65	(0.55, 0.76)
SOD (mmol/L)	0.60	(0.54, 0.69)	0.61	(0.52, 0.73)
TNF‐*α* (pg/mL)	0.59	(0.52, 0.67)	0.59	(0.50, 0.72)
IL‐6 (pg/mL)	0.60	(0.54, 0.69)	0.62	(0.50, 0.73)
CRP (mg/L)	0.64	(0.60, 0.72)	0.65	(0.53, 0.76)
ALT (U/L)	0.65	(0.60, 0.71)	0.62	(0.52, 0.72)
AST (U/L)	0.61	(0.56, 0.70)	0.60	(0.52, 0.68)

**Table 2:** Statistical models of clinical outcomes.

Outcomes	Heterogeneity		Analysis	Summary statistic
	*I* ^2^ (%)	*p* value		
BMI (kg/m^2^)	0	0.03	Fixed	MD
WC (cm)	13	0.02	Fixed	MD
WHR (mmol/L)	100	0.44	Random	MD
Leptin (*μ*g/L)	69	0.19	Random	SMD
FM (kg)	78	0.51	Random	SMD
FBG (*μ*g/dL)	45	<0.00001	Fixed	SMD
HOMAir	48	<0.00001	Fixed	MD
TC (*μ*g/dL)	82	0.04	Random	SMD
TG (*μ*g/dL)	0	0.01	Fixed	SMD
LDL (*μ*g/dL)	0	0.03	Fixed	SMD
HDL (*μ*g/dL)	62	0.03	Random	SMD
MDA (mmol/L)	84	0.001	Random	SMD
SOD (mmol/L)	54	0.0002	Random	SMD
TNF‐*α* (pg/mL)	31	0.03	Fixed	SMD
IL‐6 (pg/mL)	0	0.05	Fixed	SMD
CRP (mg/L)	0	0.86	Random	SMD
ALT (U/L)	0	0.34	Fixed	MD
AST (U/L)	0	0.55	Random	MD

BMI: body mass index; WC: waist circumference; WHR: waist-to-hip ratio; FM: fat mass; FBG: fasting plasma glucose; HOMA-ir: homeostasis model assessment of insulin resistance; TC: total cholesterol; TG: triglycerides; LDL: low-density lipoprotein; HDL: high-density lipoprotein; MDA: malondialdehyde; SOD: superoxide dismutase; TNF‐*α*: tumor necrosis factor‐*α*; IL-6: interleukin-6; CRP: C-reactive protein; ALT: alanine transaminase; AST: aspartate transaminase.

**Table 3:** Characteristics of included studies.

Study	Country	Sample size (antioxidant/control)	Population characteristic (antioxidant/control)	Intervention (antioxidant/control)	Subgroup on antioxidants	Control group	Duration of intervention
Caballero [1]	UK	36/78	Age(years):42.9 ± 7.1/42.8 ± 7.2 BMI (kg/m^2^): 30.0 ± 4.4/30.3 ± 3.5	The Ascophyllum (poly)phenol-rich blend 400 mg/day	Water-soluble antioxidants	Placebo	8 weeks
Collaborators et al. [2]	Poland	30/30	Age (years): 43.8 ± 8.2/41.0 ± 8.8 BMI (kg/m^2^): 39.2 ± 6.0/37.5 ± 4.8	Average arginine 43.3 mg/kg/day bodyweight in women and 48.6 mg/kg/day in men	Water-soluble antioxidants	Placebo	3 months
Bazrafshani et al. [3]	Iran	16/19	Age (years): 38 ± 10.9/42 ± 14.4 BMI (kg/m^2^): 37.1 ± 8.9 41.8 ± 8.5	L-selenomethionine (S) 240 *μ*g/day	Water-soluble antioxidants	Placebo	12 weeks
Valerio et al. [4]	Iran	27/28	Age (years): 44.07 ± 7.82/42.39 ± 7.21 BMI (kg/m^2^): 33.16 ± 1.72/35.03 ± 3.48	L-arginine 6 g/d	Water-soluble antioxidants	Placebo	8 weeks
Malone and Hansen [5]	Brazil	24/22	BMI (kg/m^2^): 32.5 ± 4.3/33.3 ± 4.6	Roasted baru almonds 20 g/day	Mixed	Placebo	8 weeks
Nikolopoulou and Kadoglou [6]	Brazil	24/22	BMI (kg/m^2^): 32.54 ± 4.35/33.34 ± 4.69	Roasted baru almonds 20 g/day	Mixed	Placebo	8 weeks
Milic et al. [7]	USA	19/19	BMI (kg/m^2^): 92.22 ± 3.52/92.75 ± 4.13	Dried apple 240 kcal/day	Mixed	Placebo	8 weeks
Fernández-Sánchez et al. [8]	Iran	25/25	Age (years): 39.44 ± 10.54/40.68 ± 9.87 BMI (kg/m^2^): 31.23 ± 3.03/31.47 ± 3.85	Zinc gluconate (contained 30 mg/d elemental zinc)	Water-soluble antioxidants	Placebo	12 weeks
Fonseca-Alaniz et al. [9]	Iran	23/22	Age (years): 40.6 ± 5.6/38.8 ± 6.5 BMI (kg/m^2^): 33.8 ± 3.7/34.1 ± 4.5	ALA 1200 mg/day plus vitamin E 400 mg/day	Fat-soluble antioxidants	Placebo	12 weeks
Dludla et al. [10]	Iran	30/30	Age (years): 39.16 ± 9.59/36.36 ± 9.9 BMI (kg/m^2^): 37.14 ± 5.40/36.29 ± 4.66	Majoun 10 g/day	Water-soluble antioxidants	Placebo	12 weeks
Bjorklund and Chirumbolo [11]	Iran	35/35	Age (years): 37.23 ± 9.34/37.00 ± 7.90 BMI (kg/m^2^) 29.24 ± 3.36/30.39 ± 4.69	Caraway seed extract 30 ml/day	Water-soluble antioxidants	Placebo	12 weeks
Ohishi et al. [12]	Iran	18/22	Age (years): 35.63 ± 3.2/32.95 ± 1.7 BMI (kg/m^2^): 33.17 ± 6.34/32.64 ± 2.37	Zinc 30 mg/day	Water-soluble antioxidants	Placebo	15 weeks
Szulinska et al. [13]	Germany	23/23	BMI (kg/m^2^): 32.8 ± 0.8/32.8 ± 0.8	Epicatechin 25 mg/day	Water-soluble antioxidants	Placebo	2 weeks
Hosseinpour-Arjmand [14]	China	30/29	Age (years): 41.2 ± 6.8/42.8 ± 6.9 BMI (kg/m^2^): 31.1 ± 2.7/30.8 ± 2.5	29 multivitamins and minerals one tablet/day	Mixed	Placebo	26 weeks
Callcott et al. [15]	Brazil	30/39	Age (years): 42.3 ± 9.1/40.4 ± 10.2 BMI (kg/m^2^): 34.2 ± 5.1/37.1 ± 7.2	HD+frozen açaí (Euterpe oleracea Mart) 200 g/day	Water-soluble antioxidants	HD+placebo	60 days
Zhao et al. [16]	Mexico	15/24	Age (years): 33.7 ± 11.9/38.8 ± 9.59 BMI (kg/m^2^): 35.6 ± 2.71/34.7 ± 2.89	Resveratrol 100 mg/day	Fat-soluble antioxidants	Placebo	24 weeks
Showell et al. [17]	Brazil	28/28	Age (years): 35.5 ± 6.5/33.9 ± 5.4 BMI (kg/m^2^): 35.8 ± 2.2/36.5 ± 2.5	Zinc aminochelate 30 mg/day	Water-soluble antioxidants	Placebo	30 days
Emami et al. [18]	Iran	29/29	Age (years): 36.0 ± 11.9/33.6 ± 4.8 BMI (kg/m^2^): 33.6 ± 4.8/32.7 ± 3.7	Dried licorice extract 0.5 g/day	Water-soluble antioxidants	Placebo	8 weeks
Ekhlasi et al. [19]	Netherlands	11/14	Age (years): 36 ± 3/40 ± 3 BMI (kg/m^2^): 30.5 ± 0.7/29.7 ± 1.1	Polyphenols epigallocatechin-gallate 282 mg/d and resveratrol 80 mg/d	Water-soluble antioxidants	Placebo	12 weeks
Shadman et al. [20]	Poland	28/28	Age (years): 49.2 ± 8.8/51.5 ± 7.4 BMI (kg/m^2^): 32.5 ± 3.3/33.9 ± 2.3	Green tea extract 1 capsule/day	Water-soluble Antioxidants	Placebo	8 weeks
Showell et al. [21]	Iran	30/30	Age (years): 31 ± 8/33 ± 8 BMI (kg/m^2^): 34.7 ± 4.3/33.3 ± 5.7	Zinc 30 mg/kg	Water-soluble antioxidants	Placebo	4 weeks
Smits et al. [22]	Poland	24/35	Age (years): 47.31 ± 12.04/45.92 ± 9.33 BMI (kg/m^2^): 34.44 ± 2.69/34.77 ± 3.00	DHA and EPA given in 3 capsules/day	Fat-soluble antioxidants	Placebo	12 weeks
Piche et al. [23]	Brazil	13/14	Age (years): 45.76 ± 2.58/45.07 ± 3.42 BMI (kg/m^2^): 34.63 ± 1.20/33.82 ± 0.71	Juçara berry (Euterpe edulis Mart.) freeze-dried pulp 5 g/day	Mixed	Placebo	6 weeks
Leisegang et al. [24]	USA	15/17	Age (years): 54 ± 3/49 ± 3 BMI (kg/m^2^): 36.8 ± 0.9/38.0 ± 0.9	Blueberry powder 45 g/day	Mixed	Placebo	8 weeks
Park et al. [25]	Poland	44/44	Age (years): 43.1 ± 8.6/41.5 ± 9.1 BMI (kg/m^2^): 36.8 ± 6.3/36.1 ± 4.9	The average arginine 43.3 mg/kg/day	Water-soluble antioxidants	Placebo	6 months
Dostal et al. [26]	Poland	15/15	Age (years): 37.7 ± 3.40/36.3 ± 4.18 BMI (kg/m^2^): 37.8 ± 1.51/38.2 ± 1.94	Melatonin 10 mg/day	Fat-soluble antioxidants	Placebo	30 days
Zhang et al. [27]	Poland	25/25	Age (years): 49.3 ± 8.7/50.2 ± 7.2 BMI (kg/m^2^): 33.5 ± 6.7/33.3 ± 6.2	Spirulina 0.5 g/day	Water-soluble antioxidants	Placebo	12 weeks
Farr et al. [28]	Poland	46/46	Age (years): 53.0 ± 5.8/53.6 ± 5.5 BMI (kg/m^2^): 30.3 (26.7–38.3)/33.0 (29.2–36.1)	Extract of garlic (2% allicin) 400 mg/d	Mixed	Placebo	12 weeks
Yadav et al. [29]	Iran	25/25	Age (years): 32.2 ± 6.9/35.1 ± 7.2 BMI (kg/m^2^): 32.3 ± 4.2/32.4 ± 5.9	Green tea 1 g/day, capsaicin 100 mg/day, and ginger 200 mg/day	Water-soluble antioxidant	Placebo	8 weeks
Balsan et al. [30]	Australia	28/28	Age (years): 61.4 ± 1.5/57.9 ± 1.4 BMI (kg/m^2^): 34.6 ± 0.7/37.0 ± 1.3	A fruit and vegetable concentrate supplement	Mixed	Placebo	8 weeks

**Table 4:** Risk of bias analysis of included trials.

Study	Randomization		Blinding of participants and personnel (performance bias)		Allocation concealment (selection bias)	Integrality of date outcome (attrition bias)	Selective reporting (reporting bias)	Other bias
Caballero [1]	Computer simulation	Random	Double-blinded simulation	Randomized	Unclear	Unclear	Unclear	Unclear
Collaborators et al. [2]	Unclear		Unclear		Unclear	Unclear	Unclear	Unclear
Bazrafshani et al. [3]	Unclear		Single-blind simulation	Randomized	Unclear	Unclear	Unclear	Unclear
Valerio et al. [4]	Unclear		Unclear		Unclear	Unclear	Unclear	Unclear
Malone and Hansen [5]	Computer simulation	Random	Double-blinded simulation	Randomized	Unclear	Unclear	Unclear	Unclear
Nikolopoulou and Kadoglou [6]	Computer simulation	Random	Double-blinded simulation	Randomized	Unclear	Unclear	Unclear	Unclear
Milic et al. [7]	Randomization list		Unclear		Unclear	Unclear	Unclear	Unclear
Fernández-Sánchez et al. [8]	Block size of 4 subjects' schedule		Double-blinded simulation	Randomized	Unclear	Unclear	Unclear	Unclear
Fonseca-Alaniz et al. [9]	Computer simulation	Random	Double-blinded simulation	Randomized	Unclear	Unclear	Unclear	Unclear
Dludla et al. [10]	Unclear		Double-blinded simulation	Randomized	Unclear	Unclear	Unclear	Unclear
Bjorklund and Chirumbolo [11]	Computer simulation	Random	Triple-blind simulation	Randomized	Unclear	Unclear	Unclear	Unclear
Ohishi et al. [12]	Blocked size of 4 number tables		Double-blinded simulation	Randomized	Unclear	Unclear	Unclear	Unclear
Szulinska et al. [13]	Blocked size of 4 number tables		Double-blinded simulation	Randomized	Unclear	Unclear	Unclear	Unclear
Hosseinpour-Arjmand [14]	Computer simulation	Random	Unclear		Unclear	Unclear	Unclear	Unclear
Callcott et al. [15]	Simulation blocked size of 4	Random	Double-blinded	Randomized	Unclear	Unclear	Unclear	Unclear
Zhao et al. [16]	Random number table		Simulation		Unclear	Unclear	Unclear	Unclear
Showell et al. [17]	Unclear		Unclear		Unclear	Unclear	Unclear	Unclear
Emami et al. [18]	Random number table with a permuted block size of two		Double-blinded simulation	Randomized	Unclear	Unclear	Unclear	Unclear
Ekhlasi et al. [19]	Unclear		Double-blinded simulation	Randomized	Unclear	Unclear	Unclear	Unclear
Shadman et al. [20]	Unclear		Double-blinded simulation	Randomized	Unclear	Unclear	Unclear	Unclear
Showell et al. [21]	Unclear		Double-blinded simulation	Randomized	Unclear	Unclear	Unclear	Unclear
Smits et al. [22]	Computer simulation		Double-blinded simulation	Randomized	Unclear	Unclear	Unclear	Unclear
Piche et al. [23]	Unclear	Random	Double-blinded simulation	Randomized	Unclear	Unclear	Unclear	Unclear
Leisegang et al. [24]	Unclear		Double-blinded simulation	Randomized	Unclear	Unclear	Unclear	Unclear
Park et al. [25]	Unclear		Double-blinded simulation	Randomized	Unclear	Unclear	Unclear	Unclear
Dostal et al. [26]	Unclear		Unclear		Unclear	Unclear	Unclear	Unclear
Zhang et al. [27]	Unclear		Double-blinded simulation	Randomized	Unclear	Unclear	Unclear	Unclear
Farr et al. [28]	Randomization list		Double-blinded simulation	Randomized	Unclear	Unclear	Unclear	Unclear
Yadav et al. [29]	Computer simulation	Random	Double-blinded simulation	Randomized	Unclear	Unclear	Unclear	Unclear
Balsan et al. [30]	Unclear		Double-blinded simulation	Randomized	Unclear	Unclear	Unclear	Unclear

## Data Availability

All the data are available upon reasonable request from the corresponding authors.

## Appendix

### A. Pubmed Search Strategy

Searched August 8, 2021.

#1 “Obesity”[MeSH Terms] OR “Overweight”[MeSH Terms] OR “Weight Gain”[MeSH Terms] OR “Body Mass Index”[MeSH Terms] (338,808)

#2 “obes∗”[Title/Abstract] OR “adipos∗”[Title/Abstract] OR “Overweight”[Title/Abstract] OR “Overweight”[Title/Abstract] OR “weight gain”[Title/Abstract] OR “Body Mass Index”[Title/Abstract] OR “BMI”[Title/Abstract] (632,372)

#3 #1 OR #2 (703609)

#4 “Antioxidants”[MeSH Terms] OR “Vitamins”[MeSH Terms] OR “Vitamin E”[MeSH Terms] OR “Ascorbic Acid”[MeSH Terms] OR “Tocopherols”[MeSH Terms] OR “Selenium”[MeSH Terms] OR “Zinc”[MeSH Terms] OR “Ubiquinone”[MeSH Terms] OR “Acetylcysteine”[MeSH Terms] OR “Carnitine”[MeSH Terms] OR “Melatonin”[MeSH Terms] OR “Glutathione”[MeSH Terms] OR “Carotenoids”[MeSH Terms] OR “Arginine”[MeSH Terms] OR “Resveratrol”[MeSH Terms] (528,347)

#5 “Antioxidants”[Title/Abstract] OR “Vitamin”[Title/Abstract] OR “vitamin E”[Title/Abstract] OR “tocopherol∗”[Title/Abstract] OR “alpha tocopherol∗”[Title/Abstract] OR “tocotrienol”[Title/Abstract] OR “vitamin C”[Title/Abstract] OR “ascorbic acid”[Title/Abstract] OR “ascorb∗”[Title/Abstract] OR “selenium”[Title/Abstract] OR “selen∗”[Title/Abstract] OR “zinc”[Title/Abstract] OR “zinc∗”[Title/Abstract] OR “ubiquinone”[Title/Abstract] OR “ubiquinol”[Title/Abstract] OR “coenzyme Q10”[Title/Abstract] OR “CoQ10”[Title/Abstract] OR “Acetylcysteine”[Title/Abstract] OR “Carnitine”[Title/Abstract] OR “carnitene”[Title/Abstract] OR “melatonin”[Title/Abstract] OR “Glutathione”[Title/Abstract] OR “GSH”[Title/Abstract] OR “carotene”[Title/Abstract] OR “betacarotene”[Title/Abstract] OR “Arginine”[Title/Abstract] OR “resveratrol∗”[Title/Abstract] (753,601)

#6 #4 OR #5 (955,211)

#7 #3 AND #6 (33091)

#8 (“Randomized Controlled Trial”[Publication Type] OR “Controlled Clinical Trial”[Publication Type] OR “Clinical Trials as Topic”[MeSH Terms:noexp] OR “randomized”[Title/Abstract] OR “placebo”[Title/Abstract] OR “randomly”[Title/Abstract] OR “trial”[Title/Abstract]) NOT(“Animals”[MeSH Terms] OR “mice”[Title/Abstract] OR “mouse”[Title/Abstract] OR “review”[Title/Abstract] OR “rats”[Title/Abstract] OR “fish”[Title/Abstract]) (169,491)

#9 #7 AND #8 (465)

### B. Embase Search Strategy

Searched August 3, 2021.

#1 “obesity'/exp OR “overweight”/exp OR “weight gain”/exp OR “body mass index”/exp (1110754)

#2 obes∗:ab,ti OR adipos∗:ab,ti OR overweight:ab,ti OR “over weight”:ab,ti OR “weight gain”:ab,ti OR “body mass index”:ab,ti OR bmi:ab,ti (971,155)

#3 #1 OR #2 (1,263,198)

#4 “antioxidants”/exp OR “vitamins”/exp OR “vitamin e”/exp OR “ascorbic acid”/exp OR “Tocopherols”/exp OR “Selenium”/exp OR “Zinc”/exp OR “Ubiquinone”/exp OR “Acetylcysteine”/exp OR “Carnitine”/exp OR “Melatonin”/exp OR “Glutathione”/exp OR “Carotenoids”/exp OR “Arginine”/exp OR “Resveratrol”/exp (1,278,012)

#5 Antioxidants:ab,ti OR Vitamin:ab,ti ORVitamin E:ab,ti OR tocopherol∗:ab,ti OR alpha tocopherol∗:ab,ti OR tocotrienol:ab,ti OR vitamin C:ab,ti OR ascorbic acid:ab,ti OR ascorb∗:ab,ti OR selenium:ab,ti OR selen∗:ab,ti OR zinc C:ab,ti OR zinc∗:ab,ti OR ubiquinone:ab,ti OR ubiquinol:ab,ti OR coenzyme Q10:ab,ti OR CoQ10:ab,ti OR Acetylcysteine:ab,ti OR Carnitine:ab,ti OR carnitene:ab,ti OR melatonin:ab,ti OR Glutathione:ab,ti OR GSH:ab,ti OR carotene:ab,ti OR betacarotene:ab,ti OR Arginine:ab,ti OR resveratrol∗:ab,ti (381,624)

#6 #4 OR #5 (1,418,211)

#7 (“randomized controlled trial”/exp OR “controlled clinical trial”/exp OR “clinical trials as topic”/exp OR “randomized”:ab,ti OR “placebo”:ab,ti OR “randomly”:ab,ti OR “trial”:ab,ti) NOT “animals”/exp NOT “mice”:ab,ti NOT “mouse”:ab,ti NOT “fish”:ab,ti NOT “review”:ab,ti (137,022)

#8 #6 AND #7 (4732)

#9 #3 AND #8(341)

((“obesity”/exp OR “overweight”/exp OR “weight gain”/exp OR “body mass index”/exp) OR (obes∗:ab,ti OR adipos∗:ab,ti OR overweight:ab,ti OR “over weight”:ab,ti OR “weight gain”:ab,ti OR “body mass index”:ab,ti OR bmi:ab,ti OR “HFD”:ab,ti)) AND ((“antioxidants”/exp OR “vitamins”/exp OR “vitamin e”/exp OR “ascorbic acid”/exp OR “Tocopherols”/exp OR “Selenium”/exp OR “Zinc”/exp OR “Ubiquinone”/exp OR “Acetylcysteine”/exp OR “Carnitine”/exp OR “Melatonin”/exp OR “Glutathione”/exp OR “Carotenoids”/exp OR “Arginine”/exp OR “Resveratrol”/exp) OR (Antioxidants:ab,ti OR Vitamin:ab,ti ORVitamin E:ab,ti OR tocopherol∗:ab,ti OR alpha tocopherol∗:ab,ti OR tocotrienol:ab,ti OR vitamin C:ab,ti OR ascorbic acid:ab,ti OR ascorb∗:ab,ti OR selenium:ab,ti OR selen∗:ab,ti OR zinc C:ab,ti OR zinc∗:ab,ti OR ubiquinone:ab,ti OR ubiquinol:ab,ti OR coenzyme Q10:ab,ti OR CoQ10:ab,ti OR Acetylcysteine:ab,ti OR Carnitine:ab,ti OR carnitene:ab,ti OR melatonin:ab,ti OR Glutathione:ab,ti OR GSH:ab,ti OR carotene:ab,ti OR betacarotene:ab,ti OR Arginine:ab,ti OR resveratrol∗:ab,ti)) AND ((“randomized controlled trial”/exp OR “controlled clinical trial”/exp OR “clinical trials as topic”/exp OR “randomized”:ab,ti OR “placebo”:ab,ti OR “randomly”:ab,ti OR “trial”:ab,ti) NOT “animals”/exp NOT “mice”:ab,ti NOT “mouse”:ab,ti NOT “fish”:ab,ti NOT “review”:ab,ti)

### C. The Cochrane Library Search Strategy

Searched August 8, 2021.

#1 MeSH descriptor:[Obesity] explode all trees(14700)

#2 MeSH descriptor:[Overweight] explode all trees(17455)

#3 MeSH descriptor:[Weight Gain] explode all trees(2668)

#4 MeSH descriptor:[Body Mass Index] explode all trees(10459)

#5 (obes∗):ti,ab,kw(45850)

#6 (adipos∗):ti,ab,kw(8227)

#7 (Overweight):ti,ab,kw(17932)

#8 (Over weight):ti,ab,kw(27326)

#9 (weight gain):ti,ab,kw(13878)

#10 (Body Mass Index):ti,ab,kw(44398)

#11 (BMI):ti,ab,kw(43349)

#12 #1 OR #2 OR #3 OR #4 OR #5 OR #6 OR #7 OR #8 OR #9 OR #10 OR #11 (122448)

#13 (Resveratrol∗):ti,ab,kw(676)

#14 (betacarotene):ti,ab,kw(1226)

#15 MeSH descriptor:[Antioxidants] explode all trees(4999)

#16 (Antioxidants):ti,ab,kw (6575)

#17 MeSH descriptor:[Vitamins] explode all trees(4863)

#18 (Vitamin):ti,ab,kw(30878)

#19 MeSH descriptor:[Vitamin E] explode all trees(2572)

#20 (“Vitamin E”):ti,ab,kw(4792)

#21 MeSH descriptor:[Tocopherols] explode all trees(752)

#22 (Tocopherol∗):ti,ab,kw(2989)

#23 (alpha tocopherol∗):ti,ab,kw(2456)

#24 (tocotrienol):ti,ab,kw(121)

#25 MeSH descriptor:[Ascorbic Acid] explode all trees(2291)

#26 (“Vitamin C”):ti,ab,kw (3631)

#27 (ascorbic acid):ti,ab,kw(3915)

#28 (ascorb∗):ti,ab,kw(4153)

#29 MeSH descriptor:[Selenium] explode all trees(736)

#30 (Selenium):ti,ab,kw (2163)

#31 (selen∗):ti,ab,kw(2459)

#32 MeSH descriptor:[Zinc] explode all trees(1677)

#33 (Zinc):ti,ab,kw(8083)

#34 MeSH descriptor:[Ubiquinone] explode all trees(576)

#35 (ubiquinone):ti,ab,kw (673)

#36 (ubiquinol):ti,ab,kw(121)

#37 (“coenzyme Q10”):ti,ab,kw(1016)

#38 (CoQ10):ti,ab,kw(506)

#39 MeSH descriptor:[Acetylcysteine] explode all trees(1155)

#40 (Acetylcysteine):ti,ab,kw(2337)

#41 MeSH descriptor:[Carnitine] explode all trees(649)

#42 (Carnitine):ti,ab,kw (1825)

#43 (carnitene):ti,ab,kw(3)

#44 MeSH descriptor:[Melatonin] explode all trees(1255)

#45 (melatonin):ti,ab,kw (3058)

#46 MeSH descriptor:[Glutathione] explode all trees(692)

#47 (Glutathione):ti,ab,kw (3873)

#48 (GSH):ti,ab,kw(1286)

#49 MeSH descriptor:[Carotenoids] explode all trees(3764)

#50 (carotene):ti,ab,kw (2013)

#51 MeSH descriptor:[Arginine] explode all trees(1494)

#52 (Arginine):ti,ab,kw (4569)

#53 MeSH descriptor:[Resveratrol] explode all trees(308)

#54 #13 OR #14 OR #15 OR #16 OR #17 OR #18 OR #19 OR #20 OR #21 OR #22 OR #23 OR #24 OR #25 OR #26 OR #27 OR #28 OR #29 OR #30 OR #31 OR #32 OR #33 OR #34 OR #35 OR #36 OR #37 OR #38 OR #39 OR #40 OR #41 OR #42 OR #43 OR #44 OR #45 OR #46 OR #47 OR #48 OR #49 OR #50 OR #51 OR #52 OR #53 (61638)

#55 #12 AND #54 (7782)

#56 MeSH descriptor:[fish] explode all trees (316)

#57 (animals):ti,ab,kw(14781)

#58 MeSH descriptor:[animals] explode all trees(614391)

#59 MeSH descriptor:[mice] explode all trees (1065)

#60 (mice):ti,ab,kw(4831)

#61 (mouse):ti,ab,kw(4831)

#62 MeSH descriptor:[mouse] explode all trees (1065)

#63 MeSH descriptor:[rats] explode all trees (1008)

#64 (rats):ti,ab,kw(2816)

#65 (fish):ti,ab,kw(6542)

#66 #56 OR #57 OR #58 OR #59 OR #60 OR #61 OR #62 OR #63 OR #64 OR #65 (625298)

#67 #55 NOT #66(4475) (Trials4471)

### D. Web of Science Search Strategy

Searched August 3, 2021.

#1 TS=(Obesity OR Overweight OR Weight Gain OR Body Mass Index) OR AB=(obes∗ OR adipos∗ OR Overweight OR Over weight OR weight gain OR Body Mass Index OR BMI) (657762)

#2 TS=(Antioxidants OR Vitamins OR Vitamin E OR Ascorbic Acid OR Tocopherols OR Selenium OR Zinc OR Ubiquinone OR Acetylcysteine OR Carnitine OR Melatonin OR Glutathione OR Carotenoids] OR Arginine OR Resveratrol) OR AB=(Antioxidants OR Vitamin OR vitamin E OR tocopherol∗ OR alpha tocopherol∗ OR tocotrienol OR vitamin C OR ascorbic acid OR ascorb∗ OR selenium OR selen∗ OR zinc OR zinc∗ OR ubiquinone OR ubiquinol OR coenzyme Q10 OR CoQ10 OR Acetylcysteine OR Carnitine OR carnitene OR melatonin OR Glutathione OR GSH OR carotene OR betacarotene OR Arginine OR resveratrol∗) (926136)

#3 (TS=(Randomized Controlled Trial OR Controlled Clinical Trial OR Clinical Trials as Topic) OR AB=(randomized OR placebo OR randomly OR trial)) NOT TS=(Animals) NOT AB=(mice) NOT AB=(mouse) NOT AB=(fish) NOT AB=(review) NOT TS=(rats) NOT TS=(rabbit) (911,209)

#4 #1 AND #2 AND #3 (4670)

(TS=(Randomized Controlled Trial OR Controlled Clinical Trial OR Clinical Trials as Topic) OR AB=(randomized OR placebo OR randomly OR trial)) NOT TS=(Animals) NOT AB=(mice) NOT AB=(mouse) NOT AB=(fish) NOT TS=(rats) NOT TS=(rabbit) NOT AB=(review) AND (TS=(Antioxidants OR Vitamins OR Vitamin E OR Ascorbic Acid OR Tocopherols OR Selenium OR Zinc OR Ubiquinone OR Acetylcysteine OR Carnitine OR Melatonin OR Glutathione OR Carotenoids] OR Arginine OR Resveratrol) OR AB=(Antioxidants OR Vitamin OR vitamin E OR tocopherol∗ OR alpha tocopherol∗ OR tocotrienol OR vitamin C OR ascorbic acid OR ascorb∗ OR selenium OR selen∗ OR zinc OR zinc∗ OR ubiquinone OR ubiquinol OR coenzyme Q10 OR CoQ10 OR Acetylcysteine OR Carnitine OR carnitene OR melatonin OR Glutathione OR GSH OR carotene OR betacarotene OR Arginine OR resveratrol∗)) AND (TS=(Obesity OR Overweight OR Weight Gain OR Body Mass Index) OR AB=(obes∗ OR adipos∗ OR Overweight OR Over weight OR weight gain OR Body Mass Index OR BMI OR HFD))

### E. Scopus Search Strategy

Searched August 3, 2021.

#1 TITLE-ABS (obes∗) OR TITLE-ABS (obesity) OR TITLE-ABS (overweight) OR TITLE-ABS (“weight gain”) OR TITLE-ABS (“body mass index”) OR TITLE-ABS (adipos∗) OR TITLE-ABS (“Over weight”) OR TITLE-ABS (BMI) (756,855)

#2 TITLE-ABS (Antioxidants) OR TITLE-ABS (Vitamin) OR TITLE-ABS (“vitamin E”) OR TITLE-ABS (tocopherol∗) OR TITLE-ABS (“alpha tocopherol∗”) OR TITLE-ABS (tocotrienol) OR TITLE-ABS (“vitamin C”) OR TITLE-ABS (“ascorbic acid”) OR TITLE-ABS (ascorb∗) OR TITLE-ABS (selenium) OR TITLE-ABS (selen∗) OR TITLE-ABS (zinc) OR TITLE-ABS (ubiquinone) OR TITLE-ABS (ubiquinol) OR TITLE-ABS (“coenzyme Q10”) OR TITLE-ABS (CoQ10) OR TITLE-ABS (Acetylcysteine) OR TITLE-ABS (Carnitine) OR TITLE-ABS (carnitene) OR TITLE-ABS (melatonin) OR TITLE-ABS (Glutathione) OR TITLE-ABS (GSH) OR TITLE-ABS (carotene) OR TITLE-ABS (betacarotene) OR TITLE-ABS (Arginine) OR TITLE-ABS (resveratrol∗) (1,413,400)

#3 (TITLE-ABS (randomized) OR TITLE-ABS (placebo) OR TITLE-ABS (randomly) OR TITLE-ABS (trial)) NOT TITLE-ABS (animals) OR TITLE-ABS (mice) OR TITLE-ABS (mouse) OR TITLE-ABS (review) (228386)

#4 #1 AND #2 AND 3 (921)

(TITLE-ABS (obes∗) OR TITLE-ABS (obesity) OR TITLE-ABS (overweight) OR TITLE-ABS (“weight gain”) OR TITLE-ABS (“body mass index”) OR TITLE-ABS (adipos∗) OR TITLE-ABS (“Over weight”) OR TITLE-ABS (“BMI”)) AND ((TITLE-ABS (randomized) OR TITLE-ABS (placebo) OR TITLE-ABS (randomly) OR TITLE-ABS (trial)) NOT TITLE-ABS (animals) OR TITLE-ABS (mice) OR TITLE-ABS (mouse) OR TITLE-ABS (review)) AND (TITLE-ABS (Antioxidants) OR TITLE-ABS (Vitamin) OR TITLE-ABS (“vitamin E”) OR TITLE-ABS (tocopherol∗) OR TITLE-ABS (“alpha tocopherol∗”) OR TITLE-ABS (tocotrienol) OR TITLE-ABS (“vitamin C”) OR TITLE-ABS (“ascorbic acid”) OR TITLE-ABS (ascorb∗) OR TITLE-ABS (selenium) OR TITLE-ABS (selen∗) OR TITLE-ABS (zinc) OR TITLE-ABS (ubiquinone) OR TITLE-ABS (ubiquinol) OR TITLE-ABS (“coenzyme Q10”) OR TITLE-ABS (CoQ10) OR TITLE-ABS (Acetylcysteine) OR TITLE-ABS (Carnitine) OR TITLE-ABS (carnitene) OR TITLE-ABS (melatonin) OR TITLE-ABS (Glutathione) OR TITLE-ABS (GSH) OR TITLE-ABS (carotene) OR TITLE-ABS (betacarotene) OR TITLE-ABS (Arginine) OR TITLE-ABS (resveratrol∗))

## Supplementary Materials

Supplementary 1 Figure S1: risk of bias plot of included studies. Figure S2: funnel plot evaluating study bias of antioxidants on basic indicators of obesity: BMI (A), WC (B), WHR (C), leptin (D), FM (E), FBG (F), and HOMA-ir (G) in obesity patients and compared with the control group. Figure S3: funnel plot evaluating study bias for the effects of antioxidants on lipid metabolism indexes: TC (A), TG (B), LDL (C), and HDL (D) in obesity patients and compared with the control group. Figure S4: funnel plot evaluating study bias for the effects of antioxidants on systemic antioxidant capacity indexes MDA (A) and SOD (B) in obesity patients and compared with the control group. Figure S5: funnel plot evaluating study bias for the effects of antioxidants on inflammatory biomarkers: TNF-*α* (a), IL-6 (b), and CRP (c) in obesity patients and compared with the control group. Figure S6: funnel plot evaluating study bias for the effects of antioxidants on liver function indexes: ALT (A) and AST (B) in obesity patients and compared with the control group.

Supplementary 2 Table S1: subgroup analysis of the effects of antioxidants on BMI, WC, WHR, leptin, FM, FBG, and HOMA-ir in obesity patients and compared with the control group. Table S2: subgroup analysis of the effects of antioxidants on TC, TG, LDL, and HDL in obesity patients and compared with the control group. Table S3: subgroup analysis of the effects of antioxidants on MDA and SOD in obesity patients and compared with the control group. Table S4: subgroup analysis of the effects of antioxidants on TNF-*α*, IL-6, and CRP in obesity patients and compared with the control group. Table S5: subgroup analysis of the effects of antioxidants on ALT and AST in obesity patients and compared with the control group.
