# Supplementary figures and images for: Effects of Antioxidant Supplementation on Metabolic Disorders in Obese Patients from Randomized Clinical Controls: A Meta-Analysis and Systematic Review
Source: Oxid Med Cell Longev. 2022 Sep 1;2022:7255413. doi: 10.1155/2022/7255413 (PMC9459443; doi:10.1155/2022/7255413)

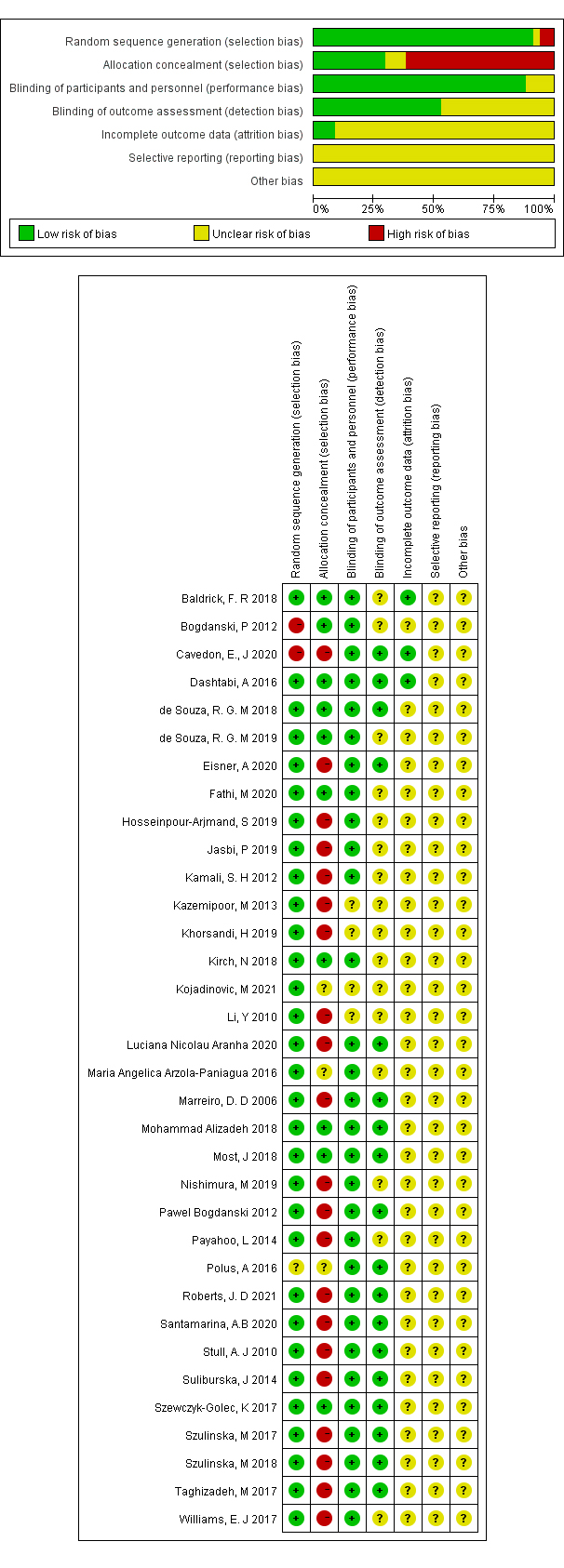

Supplement: Supplementary 1 — Figure S1: risk of bias plot of included studies. Figure S2: funnel plot evaluating study bias of antioxidants on basic indicators of obesity: BMI (A), WC (B), WHR (C), leptin (D), FM (E), FBG (F), and HOMA-ir (G) in obesity patients and compared with the control group. Figure S3: funnel plot evaluating study bias for the effects of antioxidants on lipid metabolism indexes: TC (A), TG (B), LDL (C), and HDL (D) in obesity patients and compared with the control group. Figure S4: funnel plot evaluating study bias for the effects of antioxidants on systemic antioxidant capacity indexes MDA (A) and SOD (B) in obesity patients and compared with the control group. Figure S5: funnel plot evaluating study bias for the effects of antioxidants on inflammatory biomarkers: TNF-α (a), IL-6 (b), and CRP (c) in obesity patients and compared with the control group. Figure S6: funnel plot evaluating study bias for the effects of antioxidants on liver function indexes: ALT (A) and AST (B) in obesity patients and compared with the control group. [file 7255413.f1.zip › Supplemental Figure S1.jpg]

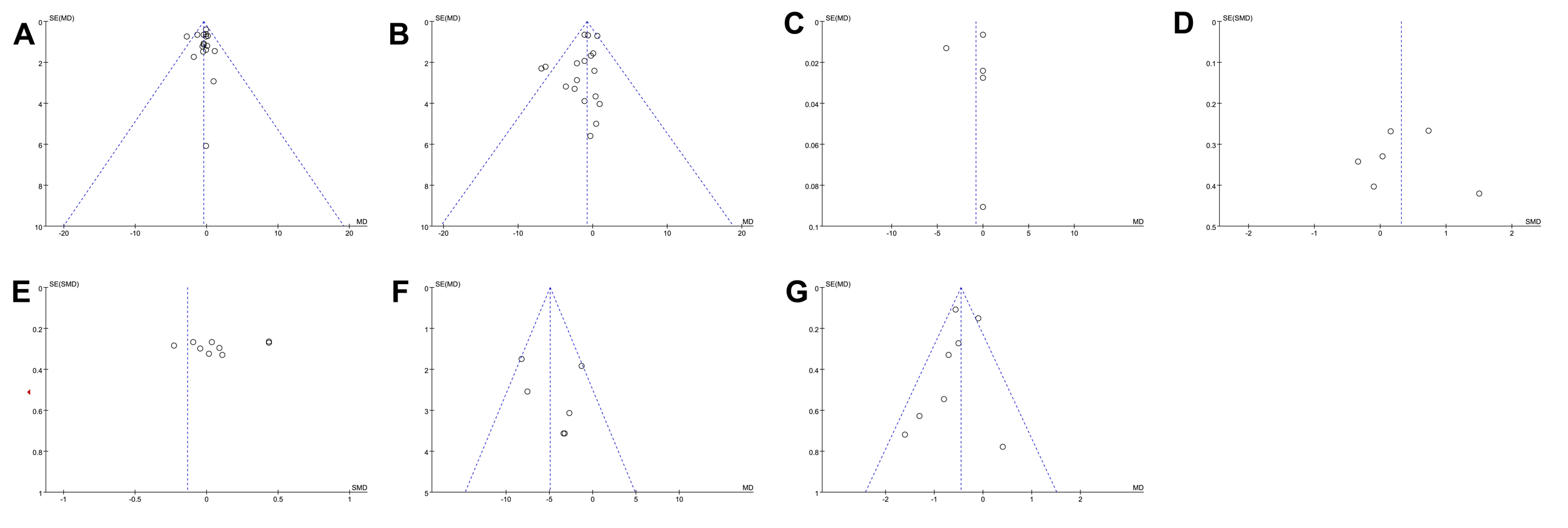

Supplement: Supplementary 1 — Figure S1: risk of bias plot of included studies. Figure S2: funnel plot evaluating study bias of antioxidants on basic indicators of obesity: BMI (A), WC (B), WHR (C), leptin (D), FM (E), FBG (F), and HOMA-ir (G) in obesity patients and compared with the control group. Figure S3: funnel plot evaluating study bias for the effects of antioxidants on lipid metabolism indexes: TC (A), TG (B), LDL (C), and HDL (D) in obesity patients and compared with the control group. Figure S4: funnel plot evaluating study bias for the effects of antioxidants on systemic antioxidant capacity indexes MDA (A) and SOD (B) in obesity patients and compared with the control group. Figure S5: funnel plot evaluating study bias for the effects of antioxidants on inflammatory biomarkers: TNF-α (a), IL-6 (b), and CRP (c) in obesity patients and compared with the control group. Figure S6: funnel plot evaluating study bias for the effects of antioxidants on liver function indexes: ALT (A) and AST (B) in obesity patients and compared with the control group. [file 7255413.f1.zip › Supplemental Figure S2.jpg]

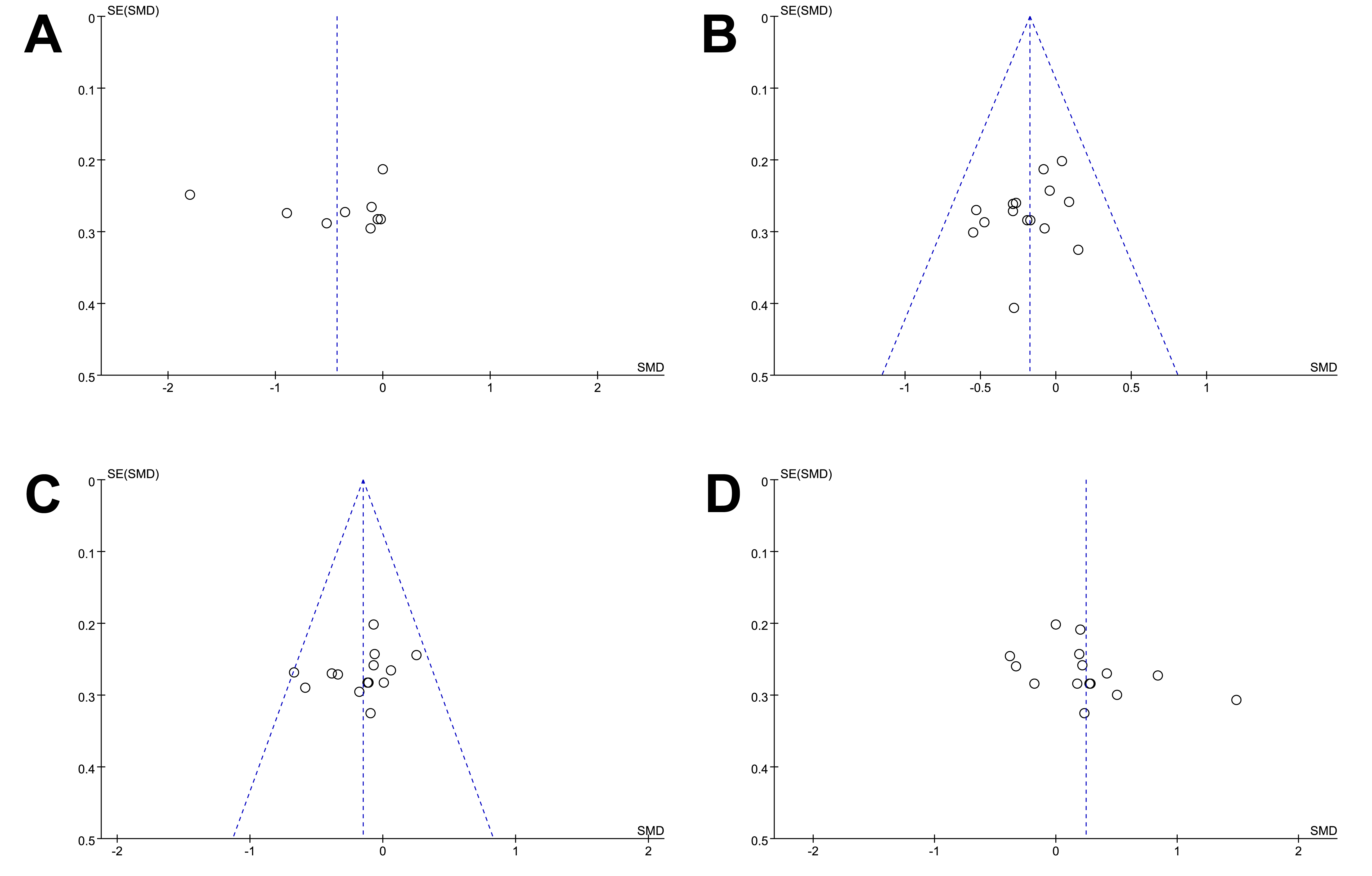

Supplement: Supplementary 1 — Figure S1: risk of bias plot of included studies. Figure S2: funnel plot evaluating study bias of antioxidants on basic indicators of obesity: BMI (A), WC (B), WHR (C), leptin (D), FM (E), FBG (F), and HOMA-ir (G) in obesity patients and compared with the control group. Figure S3: funnel plot evaluating study bias for the effects of antioxidants on lipid metabolism indexes: TC (A), TG (B), LDL (C), and HDL (D) in obesity patients and compared with the control group. Figure S4: funnel plot evaluating study bias for the effects of antioxidants on systemic antioxidant capacity indexes MDA (A) and SOD (B) in obesity patients and compared with the control group. Figure S5: funnel plot evaluating study bias for the effects of antioxidants on inflammatory biomarkers: TNF-α (a), IL-6 (b), and CRP (c) in obesity patients and compared with the control group. Figure S6: funnel plot evaluating study bias for the effects of antioxidants on liver function indexes: ALT (A) and AST (B) in obesity patients and compared with the control group. [file 7255413.f1.zip › Supplemental Figure S3.jpg]

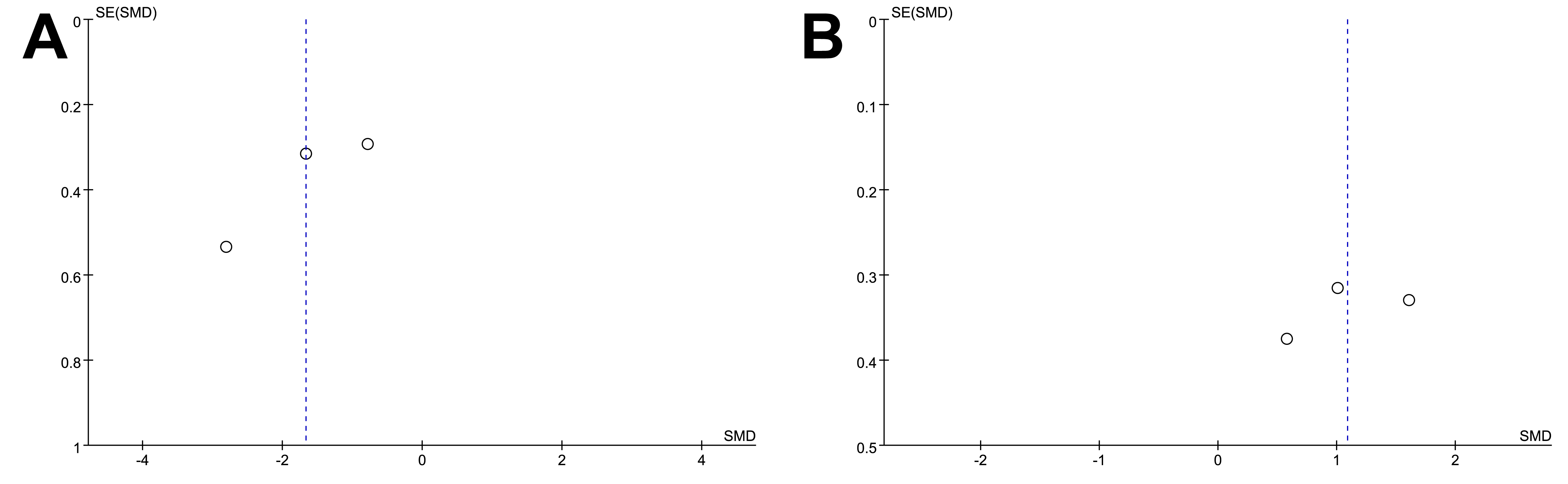

Supplement: Supplementary 1 — Figure S1: risk of bias plot of included studies. Figure S2: funnel plot evaluating study bias of antioxidants on basic indicators of obesity: BMI (A), WC (B), WHR (C), leptin (D), FM (E), FBG (F), and HOMA-ir (G) in obesity patients and compared with the control group. Figure S3: funnel plot evaluating study bias for the effects of antioxidants on lipid metabolism indexes: TC (A), TG (B), LDL (C), and HDL (D) in obesity patients and compared with the control group. Figure S4: funnel plot evaluating study bias for the effects of antioxidants on systemic antioxidant capacity indexes MDA (A) and SOD (B) in obesity patients and compared with the control group. Figure S5: funnel plot evaluating study bias for the effects of antioxidants on inflammatory biomarkers: TNF-α (a), IL-6 (b), and CRP (c) in obesity patients and compared with the control group. Figure S6: funnel plot evaluating study bias for the effects of antioxidants on liver function indexes: ALT (A) and AST (B) in obesity patients and compared with the control group. [file 7255413.f1.zip › Supplemental Figure S4.jpg]

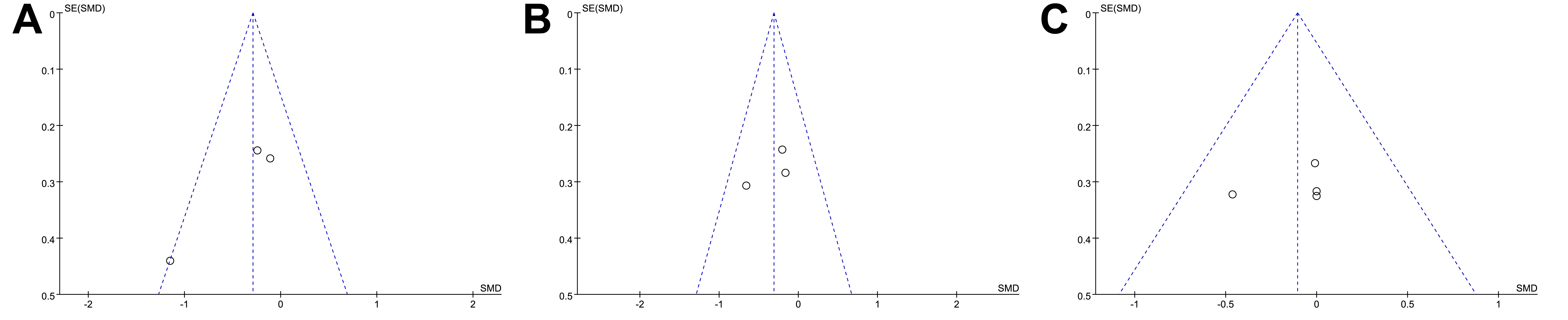

Supplement: Supplementary 1 — Figure S1: risk of bias plot of included studies. Figure S2: funnel plot evaluating study bias of antioxidants on basic indicators of obesity: BMI (A), WC (B), WHR (C), leptin (D), FM (E), FBG (F), and HOMA-ir (G) in obesity patients and compared with the control group. Figure S3: funnel plot evaluating study bias for the effects of antioxidants on lipid metabolism indexes: TC (A), TG (B), LDL (C), and HDL (D) in obesity patients and compared with the control group. Figure S4: funnel plot evaluating study bias for the effects of antioxidants on systemic antioxidant capacity indexes MDA (A) and SOD (B) in obesity patients and compared with the control group. Figure S5: funnel plot evaluating study bias for the effects of antioxidants on inflammatory biomarkers: TNF-α (a), IL-6 (b), and CRP (c) in obesity patients and compared with the control group. Figure S6: funnel plot evaluating study bias for the effects of antioxidants on liver function indexes: ALT (A) and AST (B) in obesity patients and compared with the control group. [file 7255413.f1.zip › Supplemental Figure S5.jpg]

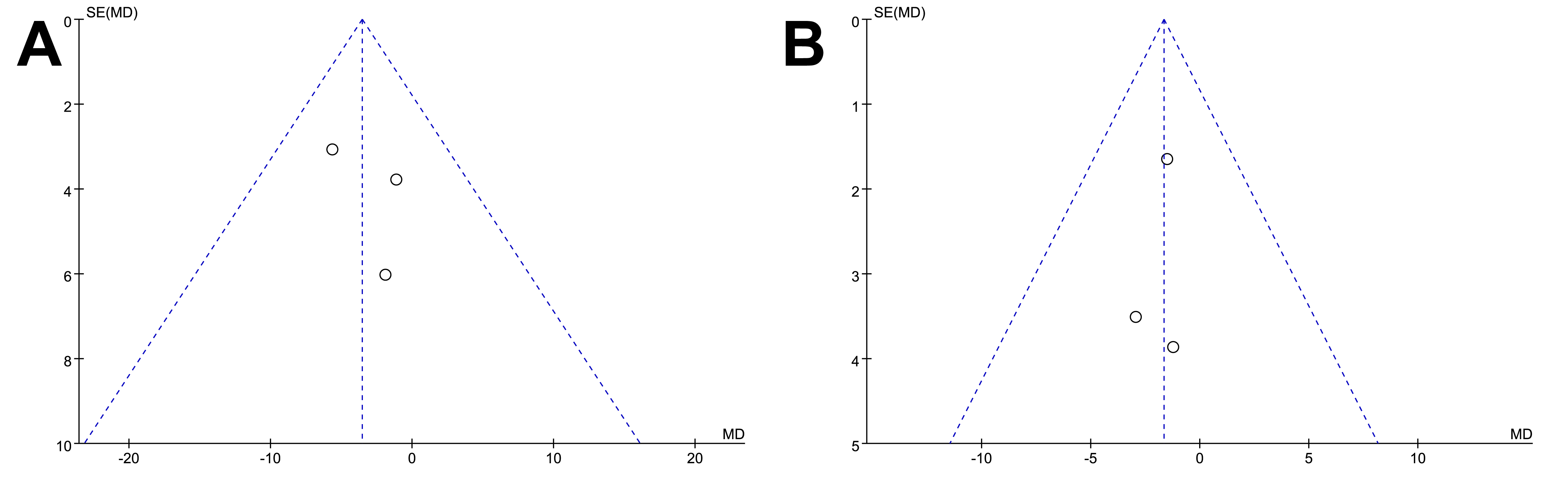

Supplement: Supplementary 1 — Figure S1: risk of bias plot of included studies. Figure S2: funnel plot evaluating study bias of antioxidants on basic indicators of obesity: BMI (A), WC (B), WHR (C), leptin (D), FM (E), FBG (F), and HOMA-ir (G) in obesity patients and compared with the control group. Figure S3: funnel plot evaluating study bias for the effects of antioxidants on lipid metabolism indexes: TC (A), TG (B), LDL (C), and HDL (D) in obesity patients and compared with the control group. Figure S4: funnel plot evaluating study bias for the effects of antioxidants on systemic antioxidant capacity indexes MDA (A) and SOD (B) in obesity patients and compared with the control group. Figure S5: funnel plot evaluating study bias for the effects of antioxidants on inflammatory biomarkers: TNF-α (a), IL-6 (b), and CRP (c) in obesity patients and compared with the control group. Figure S6: funnel plot evaluating study bias for the effects of antioxidants on liver function indexes: ALT (A) and AST (B) in obesity patients and compared with the control group. [file 7255413.f1.zip › Supplemental Figure S6.jpg]
