# Supplementary material for: Effects of Antioxidant Supplementation on Metabolic Disorders in Obese Patients from Randomized Clinical Controls: A Meta-Analysis and Systematic Review
Source: Oxid Med Cell Longev. 2022 Sep 1;2022:7255413. doi: 10.1155/2022/7255413 (PMC9459443; doi:10.1155/2022/7255413)
Supplement: Supplementary 2 — Table S1: subgroup analysis of the effects of antioxidants on BMI, WC, WHR, leptin, FM, FBG, and HOMA-ir in obesity patients and compared with the control group. Table S2: subgroup analysis of the effects of antioxidants on TC, TG, LDL, and HDL in obesity patients and compared with the control group. Table S3: subgroup analysis of the effects of antioxidants on MDA and SOD in obesity patients and compared with the control group. Table S4: subgroup analysis of the effects of antioxidants on TNF-α, IL-6, and CRP in obesity patients and compared with the control group. Table S5: subgroup analysis of the effects of antioxidants on ALT and AST in obesity patients and compared with the control group. [file 7255413.f2.pdf]

Supplemental Figure S1

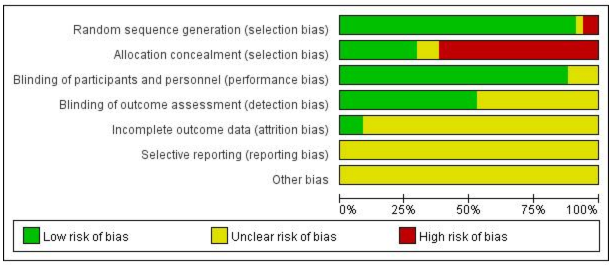

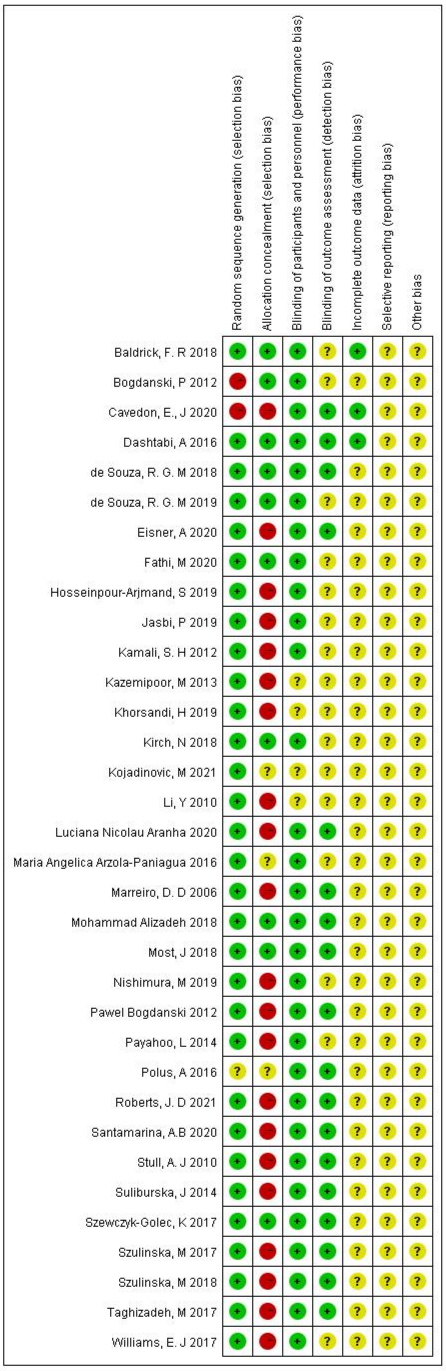

Supplemental Figure S2

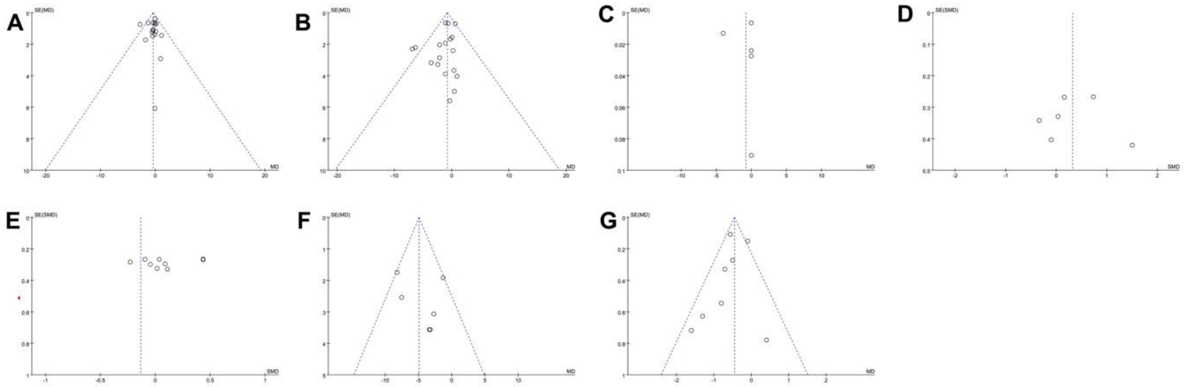

Supplemental Figure S3

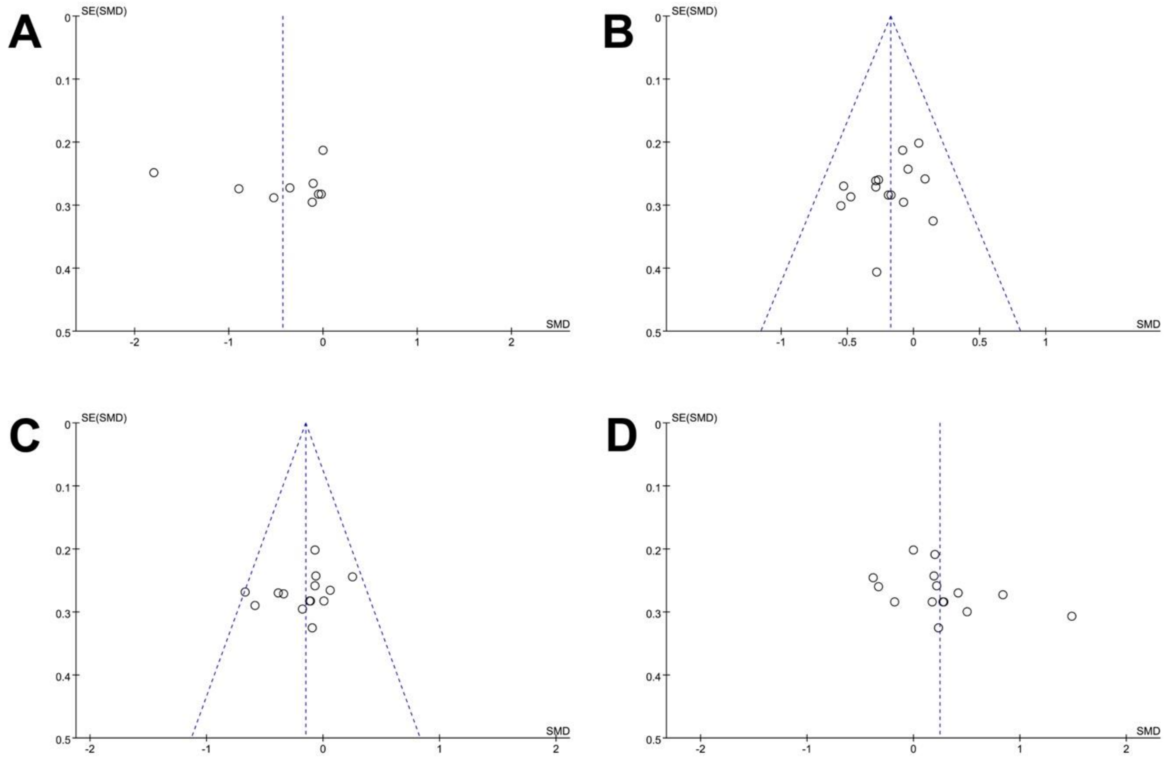

Supplemental Figure S4

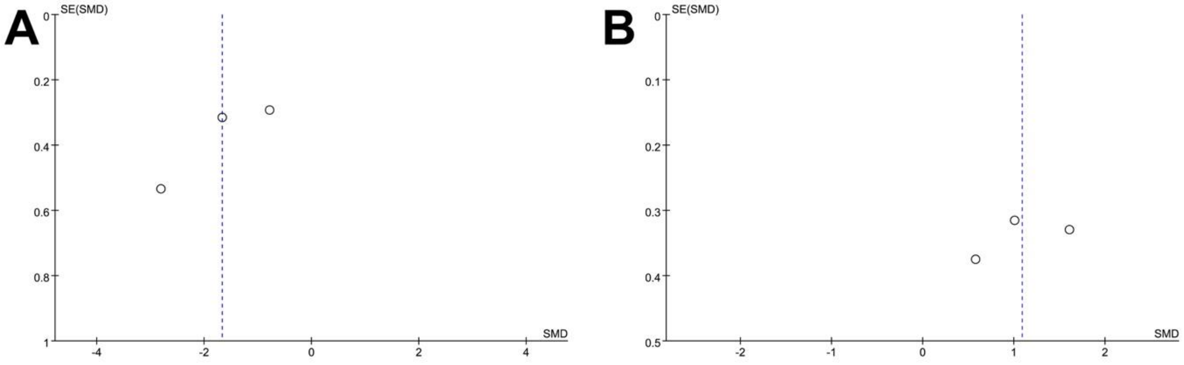

Supplemental Figure S5

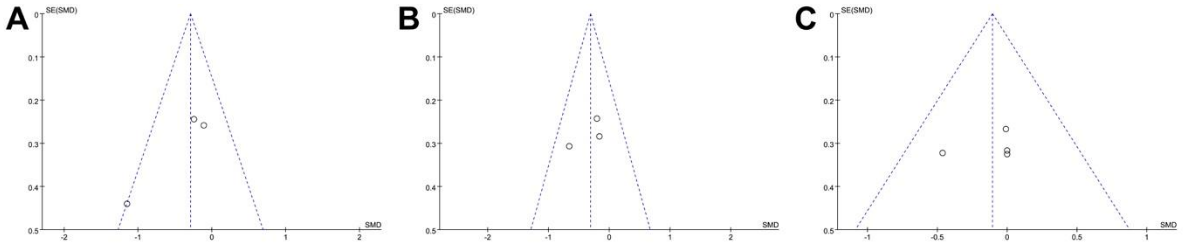

Supplemental Figure S6

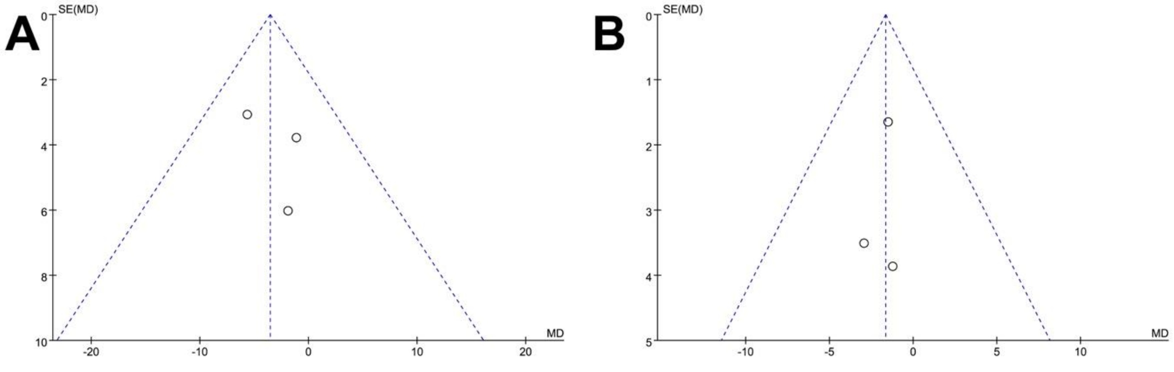

Supplementary Table S1 Subgroup analysis of the effects of antioxidants on BMI, WC, WHR, Leptin, FM, FBG and HOMA-ir in obesity patients and compared with controls group

Subgroup	BMI (kg/m^2)	WC (cm)	WHR (mmol/L)	Leptin (μg/L)	FM (kg)	FBG (μg/dL)	HOMA-ir
Water-soluble antioxidants	MD=-0.37<br>95% CI=(-0.83, 0.09),<br>P=0.12	MD=-1.00<br>95% CI=(-3.58, -1.59)<br>P=0.003	MD=-1.51<br>95% CI=(-0.63, -0.03)<br>P=0.45	SMD=0.16<br>95% CI=(-0.32, -063)<br>P=0.51	MD=-0.78<br>95% CI=(-2.36, 0.80)<br>P=0.34	SMD=-5.07<br>95% CI=(-7.09,-3.05)<br>P＜0.00001	MD=-0.46<br>95% CI=(-0.62, -0.31)<br>P＜0.00001
Fat-soluble antioxidants	MD=-0.15<br>95% CI=(-0.97,-0.68)<br>P=0.72	MD=0.37<br>95% CI=(-0.89, -1.63)<br>P=0.57	-	MD=0.74<br>95% CI=(-0.69, 2.18)<br>P=0.03	MD=0.03<br>95% CI=(-0.41, 0.46)<br>P=0.91	MD=-3.20<br>95% CI=(-10.19, 3.79)<br>P=0.37	-
Mixed antioxidants	MD=-1.12<br>95% CI=(-2.27,-0.03)<br>P=0.06	MD=-0.79<br>95% CI=(-2.04, -0.45)<br>P=0.21	MD=0.01<br>95% CI=(-0.00, 0.02)<br>P=0.13	-	SMD=0.05<br>95% CI=(-0.20, -0.30)<br>P=0.67	-	MD=0.40<br>95% CI=(-1.13, 1.93)<br>P=0.61

Supplementary Table S2 Subgroup analysis of the effects of antioxidants on TC, TG, LDL and HDL in obesity patients and compared with controls group

Subgroup	TC (μg/dL)	TG (μg/dL)	LDL (μg/dL)	HDL (μg/dL)
Water-soluble antioxidants	SMD=-0.16<br>95%CI=(-0.39, 0.07)<br>P=0.16	SMD=-0.11<br>95%CI=(-0.26, 0.05)<br>P=0.17	SMD=-0.13<br>95%CI=(-0.29, 0.04)<br>P=0.13	SMD=0.17<br>95%CI=(-0.13, -0.47)<br>P=0.26
Fat-soluble antioxidants	SMD=-0.11<br>95%CI=(-0.63, 0.41)<br>P=0.69	SMD=-0.53<br>95%CI=(-1.05, 0.00)<br>P=0.05	MD=0.06<br>95%CI=(-0.46, 0.58)<br>P=0.82	-
Mixed antioxidants	SMD=-0.94<br>95%CI=(-1.89, 0.01)<br>P=0.05	SMD=-0.19<br>95%CI=(-0.48, 0.09)<br>P=0.18	SMD=-0.28<br>95%CI=(-0.57, 0.00)<br>P=0.05	SMD=0.39<br>95%CI=(0.16, 0.63)<br>P=0.001

Supplementary Table S3 Subgroup analysis of the effects of antioxidants on MDA and SOD in obesity patients and compared with controls group

Subgroup	MDA (mmol/L)	SOD (mmol/L)
Water-soluble antioxidants	MD=-1.21<br>95%CI=(-2.08, -0.34)<br>P=0.007	MD=1.61<br>95%CI=(0.96, 2.25)<br>P＜0.00001
Fat-soluble antioxidants	MD=-2.80<br>95%CI=(-3.84, -1.76)<br>P＜0.00001	MD=0.58,<br>95%CI=(-0.15, 1.32)<br>P=0.12
Mixed antioxidants	-	MD=1.01<br>95%CI=(0.39, 1.63)<br>P=0.001

Supplementary Table S4 Subgroup analysis of the effects of antioxidants on TNF-α, IL-6 and CRP in obesity patients and compared with controls group

Subgroup	TNF-α (pg/mL)	IL-6 (pg/mL)	CRP (mg/L)
Water-soluble antioxidants	MD=-0.29 95%CI=(-0.56, -0.02) P=0.03	MD=-0.19 95%CI=(-0.55, 0.18) P=0.31	MD=-0.19 95%CI=(-0.60, 0.21) P=0.35
Fat-soluble antioxidants	-	SMD=-0.66, 95%CI=(-1.26, -0.06) P=0.03	MD=0.00, 95%CI=(-0.62, 0.62) P=1.00
Mixed antioxidants	-	-	MD=0.00 95%CI=(-0.64, 0.64) P=1.00

Supplementary Table S5 Subgroup analysis of the effects of antioxidants on ALT and AST in obesity patients and compared with controls group

Subgroup	ALT (U/L)	AST (U/L)
Water-soluble antioxidants	MD=-1.31<br>95%CI=(-4.11, 1.49)<br>P=0.36	MD=-0.32<br>95%CI=(-2.13, 1.48)<br>P=0.73
Fat-soluble antioxidants	MD=-1.90<br>95%CI=(-13.70,9.90)<br>P=0.75	MD=-2.90<br>95%CI=(-9.77,-3.97)<br>P=0.41
Mixed antioxidants	-	MD=-1.20<br>95%CI=(-8.78,6.38)<br>P=0.76
